# Comparison of Nonheme Manganese- and Iron-Containing Flavone Synthase Mimics

**DOI:** 10.3390/molecules26113220

**Published:** 2021-05-27

**Authors:** Dóra Lakk-Bogáth, Natalija Pantalon Juraj, Bashdar I. Meena, Berislav Perić, Srećko I. Kirin, József Kaizer

**Affiliations:** 1 Research Group of Bioorganic and Biocoordination Chemistry, University of Pannonia, H-8201 Veszprém, Hungary; lakkd@almos.uni-pannon.hu (D.L.-B.); bashdarismael@gmail.com (B.I.M.); 2 Division of Materials Chemistry, Ruđer Bošković Institute, Bijenička c. 54, HR-10000 Zagreb, Croatia; Natalija.Pantalon.Juraj@irb.hr (N.P.J.); Berislav.Peric@irb.hr (B.P.); srecko.kirin@irb.hr (S.I.K.)

**Keywords:** flavone synthase, iron(IV)-oxo, manganese(IV)-oxo, oxidation, kinetic studies

## Abstract

Heme and nonheme-type flavone synthase enzymes, FS I and FS II are responsible for the synthesis of flavones, which play an important role in various biological processes, and have a wide range of biomedicinal properties including antitumor, antimalarial, and antioxidant activities. To get more insight into the mechanism of this curious enzyme reaction, nonheme structural and functional models were carried out by the use of mononuclear iron, [Fe^II^(CDA-BPA*)]^2+^ (**6**) [CDA-BPA = *N*,*N*,*N*’,*N*’-*tetrakis*-(2-pyridylmethyl)-cyclohexanediamine], [Fe^II^(CDA-BQA*)]^2+^ (**5**) [CDA-BQA = *N*,*N*,*N*’,*N*’-*tetrakis*-(2-quinolilmethyl)-cyclohexanediamine], [Fe^II^(Bn-TPEN)(CH_3_CN)]^2+^ (**3**) [Bn-TPEN = *N*-benzyl-*N*,*N*’,*N*’-tris(2-pyridylmethyl)-1,2-diaminoethane], [Fe^IV^(O)(Bn-TPEN)]^2+^ (**9**), and manganese, [Mn^II^(N4Py*)(CH_3_CN)]^2+^ (**2**) [N4Py* = *N*,*N*-bis(2-pyridylmethyl)-1,2-di(2-pyridyl)ethylamine)], [Mn^II^(Bn-TPEN)(CH_3_CN)]^2+^ (**4**) complexes as catalysts, where the possible reactive intermediates, high-valent Fe^IV^(O) and Mn^IV^(O) are known and well characterised. The results of the catalytic and stoichiometric reactions showed that the ligand framework and the nature of the metal cofactor significantly influenced the reactivity of the catalyst and its intermediate. Comparing the reactions of [Fe^IV^(O)(Bn-TPEN)]^2+^ (**9**) and [Mn^IV^(O)(Bn-TPEN)]^2+^ (**10**) towards flavanone under the same conditions, a 3.5-fold difference in reaction rate was observed in favor of iron, and this value is three orders of magnitude higher than was observed for the previously published [Fe^IV^(O)(N2Py2Q*)]^2+^ [*N*,*N*-bis(2-quinolylmethyl)-1,2-di(2-pyridyl)ethylamine] species.

## 1. Introduction

**Scheme 1:**
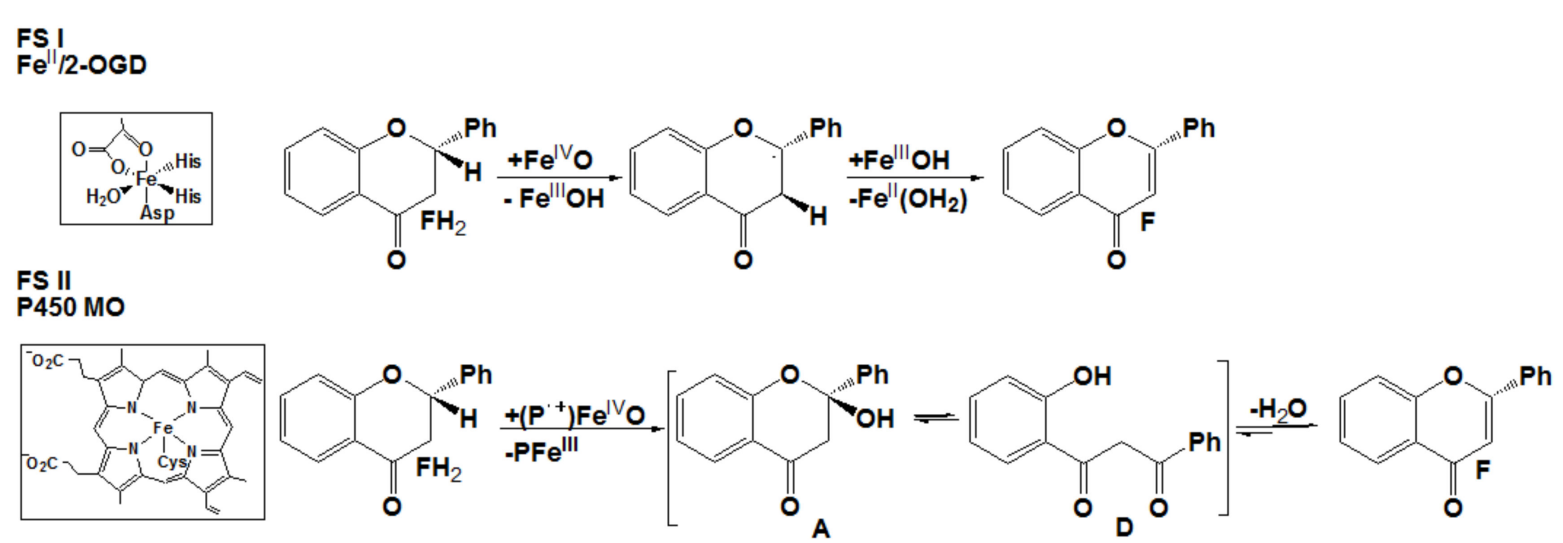
Oxidation of flavanone by heme and nonheme flavone synthases, FS I and FS II.

Flavones are low molecular weight phytochemicals that play an important role in various biological processes and have a positive impact on our health [1]. Due to their wide range of biological activities (malaria, anti-cancer, anti-diabetes, asthma, antiviral, antioxidant, anti-microbial, anti-ulcer, anti-inflammation, cardiovascular activity, neuroprotection, etc.) their syntheses have become important goals of medicinal and bioorganic chemists [2,3,4,5,6,7,8]. Flavones can be synthesised by various methods including Baker-Venkataraman-rearrangement from *o*-hydroxyacetophenone [1], and oxidation of flavanones using various stoichiometric reagents such as DMSO/I_2_ [9], SeO_2_ [10], 2,3-dichloro-5,6-dicyano-1,4-benzoquinone [11], thallium salts [12] and manganese acetate [13]. The oxidation of flavanones by heme and nonheme iron-dependent enzymes is one of the most important steps during the biosynthesis of flavones. High-valent oxoiron(IV) intermediates as key oxidants are well-established in both heme and nonheme enzymes, including cytochrome P450, bovine liver catalase (BLC) [14,15], flavone synthase II (FS II) [16,17,18,19,20], pterin-dependent phenylalanine hydroxylase [21], and α-keto acid-dependent dioxygenases (taurine dioxygenase, TauD [22,23,24] and flavone synthase I, FS I) [25,26,27,28], and their synthetic biomimetic models. Interestingly, two main classes of flavone synthase enzymes are known, FS I and FS II, with totally different active sites and catalytic mechanisms (Scheme 1). The majority of flavone synthase enzymes (FS II) contain iron(III)-protoporphyrin (PFe^III^) as a prosthetic group with (P^+•^)Fe^IV^=O oxidant (CYP93B), and the reaction proceeds via the formation of 2-hydroxyflavanone (monooxygenase activity) and its subsequent dehydration into the flavones [29]. In contrast, FS I enzymes utilise nonheme mononuclear iron(II)-2-oxoglutarate (Fe^II^/2-OG) as a prosthetic group where the reaction can be described by oxoiron(IV) mediated, direct non-concerted 2,3-desaturation without 2-hydroxyflavanone formation [30].

Since flavanone itself is a chiral molecule, oxidative kinetic resolution (OKR) of racemic flavanones can also be performed with a chiral iron catalyst and oxoiron(IV) intermediates. Increasing interest in the region and stereoselective metal-based reactions to generate new stereogenic centres in a highly diastereoselective and/or enantioselective fashion inspires the search for biomimetic oxidation catalysts. Intermediates of this type were observed in catalytic oxidation systems and synthetised and identified indirectly by the use of iron precursor complexes with various chiral and achiral aminopyridine ligands [31,32,33,34,35,36].

**Scheme 2:**
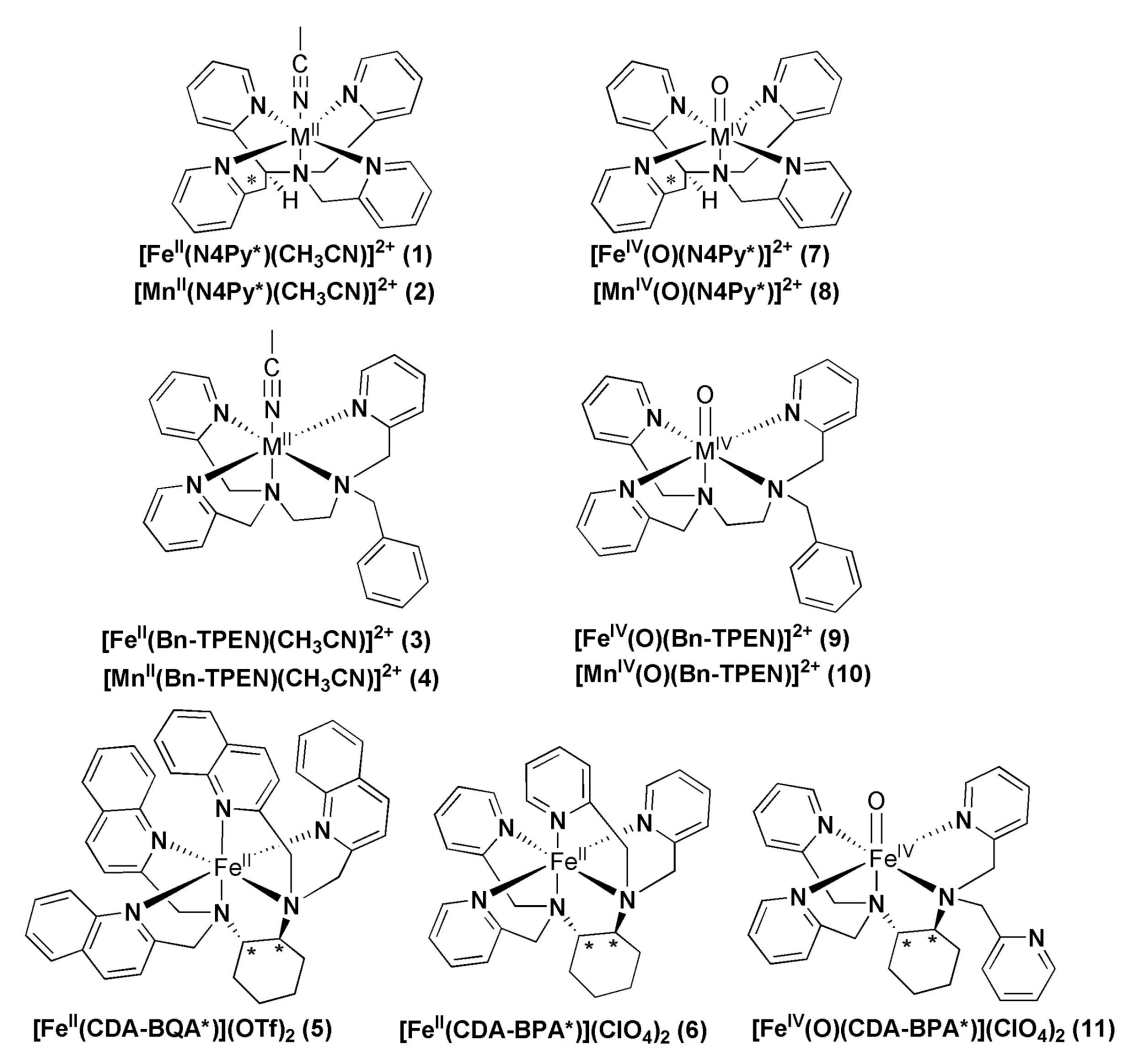
Oxoiron(IV) and oxomanganese(IV) complexes with their iron(II) and manganese(II) precursor complexes were used in this study.

In the present work, we carried out stoichiometric and catalytic flavanone oxidation reactions with spectroscopically well-characterised nonheme oxoiron(IV) intermediates compared to their analogous oxomanganese(IV) compounds, [Fe^IV^(O)(Bn-TPEN)]^2+^ (**9**) [37,38], [Fe^IV^(O)(CDA-BPA)]^2+^ (**11**), [Mn^IV^(O)(N4Py*)]^2+^ (**8**) [39], [Mn^IV^(O)(Bn-TPEN)]^2+^ (**10**) [40] and their precursor complexes, [Fe^II^(Bn-TPEN)(CH_3_CN)]^2+^ (**3**), [Fe^II^(CDA-BQA*)]^2+^ (**5**), [Fe^II^(CDA-BPA*)]^2+^ (**6**) [41], [Mn^II^(N4Py*)(CH_3_CN)]^2+^ (**2**) [39], [Mn^II^(Bn-TPEN)(CH_3_CN)]^2+^ (**4**) (Scheme 2) [40]. To the best of our knowledge, this study provides the first mechanistic details of oxomanganese(IV)-mediated flavanone oxidation compared to their analogous oxoiron(IV)-mediated systems, which may serve as a functional model of FS enzymes. Based on the detected intermediary products, the catalysis of double-bond formation is suggested to take place in two steps, namely by the monohydroxylation of the substrate, and then the elimination of water from the intermediary 2-hydroxyflavanone. This mechanism is different from the hitherto known FS I enzyme, but it is consistent with other 2-oxoglutarate-dependent enzymes, and the heme iron-dependent flavone synthase II.

## 2. Results and Discussion

### 2.1. Nonheme Iron and Manganese-Containing Biomimics of the Flavone Synthase Enzyme

The use of well-chosen ligands made it possible to prepare, spectroscopically characterise, and study the reactivity of the putative intermediates in enzymatic processes. In the last 20 years, a number of precursor iron(II) complexes with their high-valent oxoiron(IV) intermediates have been prepared by the use of multidentate N-donor ligands such as TPA, N4Py, Py5 [2,6-(bis-(bis-2-pyridyl)methoxymethane)pyridine] and TPEN (*N,*N*,*N*′,*N**′-tetrakis(2-pyridylmethyl)- ethylenediamine) [37,38,39,40,41,42]. These complexes as catalysts and/or intermediates catalyse all types of oxidation reactions such as epoxidations, heteroatom oxidations, and even C-H oxidations including hydrogen-atom transfer (HAT) and oxygen-atom transfer (OAT) [43,44,45].

The primary aspect of ligand selection, as a continuation of our previous work, was to increase the reactivity of the catalyst and its intermediates towards flavanone [46]. For this purpose, new ([Fe^II^(CDA-BQA*)]^2+^ (**5**), [Fe^IV^(O)(CDA-BPA^*^)]^2+^ (**11**)) and spectroscopically well-characterised ([Fe^II^(CDA-BPA*)]^2+^ (**6**), [Fe^II^(Bn-TPEN)(CH_3_CN)]^2+^ (**3**) [Fe^IV^(O)(Bn-TPEN)]^2+^ (**9**)) nonheme iron(II) and oxoiron(IV) complexes, were chosen. Since nonheme oxomanganese (IV) complexes have proven to be versatile oxidants [40], in addition to iron-containing models we also aimed to elucidate the role of the metal cofactor through the comparison of well-defined iron- and manganese-containing systems. Previously reported [Mn^II^(N4Py*)(CH_3_CN)]^2+^ (**2**), [Mn^II^(Bn-TPEN)(CH_3_CN)]^2+^ (**4**) as catalysts and [Mn^IV^(O)(N4Py*)]^2+^ (**8**), [Mn^IV^(O)(Bn-TPEN)]^2+^ (**10**) as possible intermediates in the oxidation reactions were chosen for these measurements [39,40]. In this work, catalytic oxidation of flavanone was performed with **2**, **3**, **4**, **5**, and **6**. Catalytic oxidation of ethylbenzene was performed with **5** and **6**, and stoichiometric oxidation reactions were performed with **7**, **8**, **9**, **10**, and **11**. *N,N,N’,N’-tetrakis*(2-pyridylmethyl)ethylenediamine (TPEN) is a well-known metal chelator. TPEN complexes are often used as Zn(II) and Cd(II) indicators, and in this case, substituting the pyridine for quinoline results in enhancement of fluorescence intensity and use of these ligands as fluorescent probes [47,48]. Fe(II) complexes of the TPEN group of ligands have interesting electronical properties, where conformational changes are linked to different spin-state interconversion processes [41]. Due to the interesting redox behaviour of these Fe(II) complexes they have been studied as superoxide dismutase mimics [49] and for their reactivity towards hydrogen peroxide [50].

**Figure 1:**
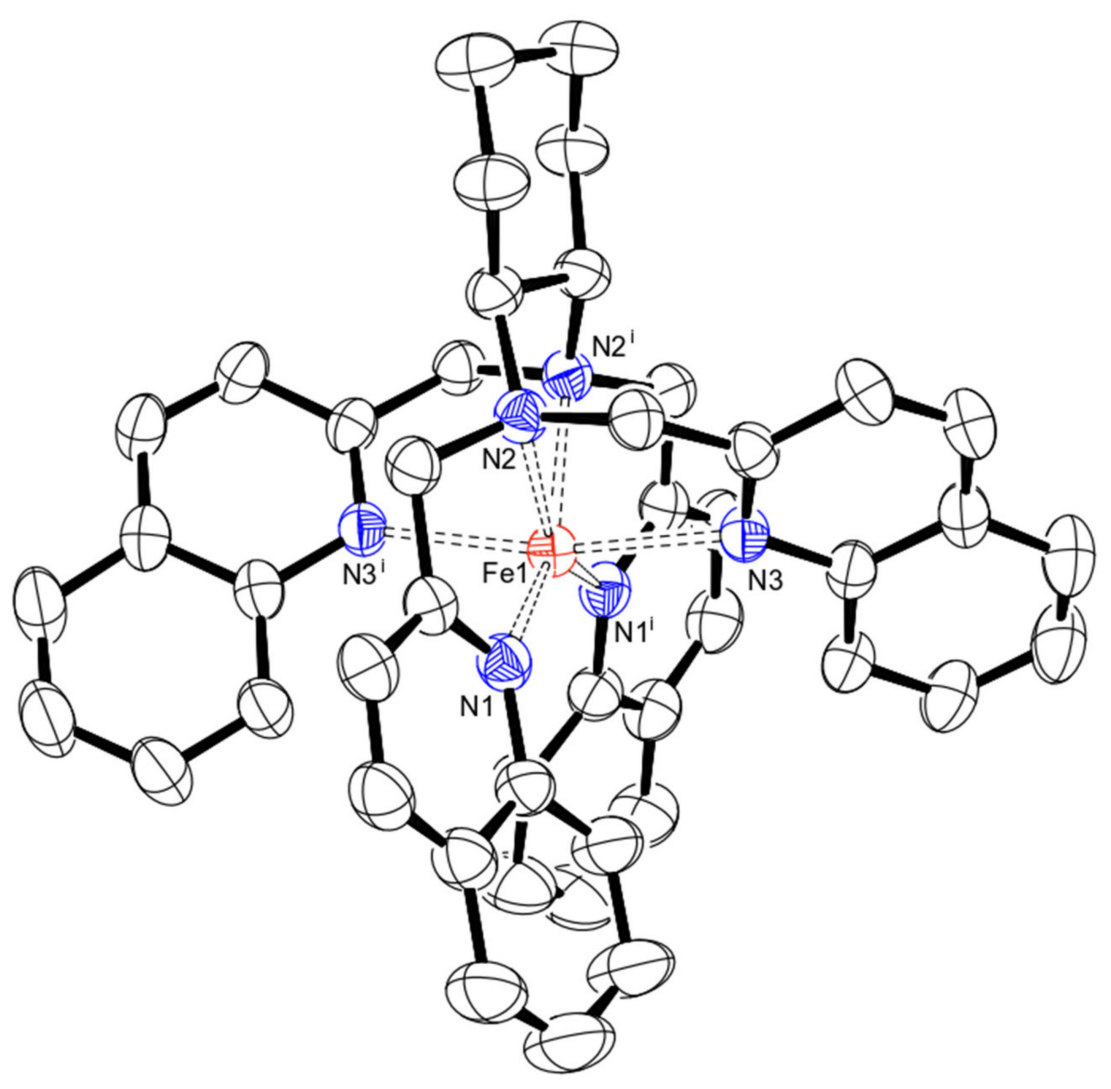
X-ray structure of Fe^II^(±CDA-BQA*)](CF_3_SO_3_)_2_ (**5**), showing a 30% probability of thermal ellipsoids.

**Figure 2:**
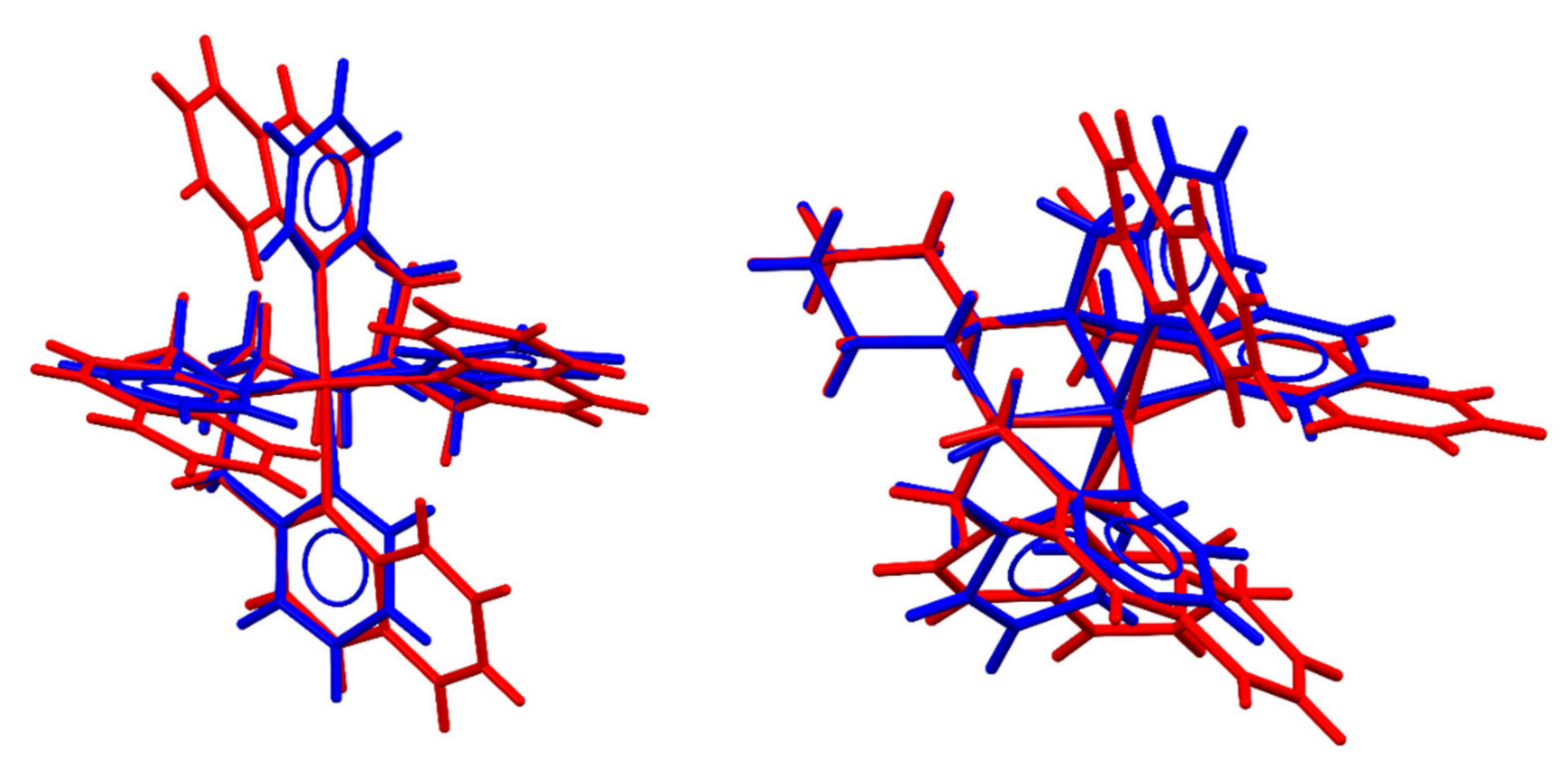
Overlay of structures Fe^II^(CDA-BQA*)](CF_3_SO_3_)_2_ (**5**) (red) and [Fe^II^(CDA-BPA*)](ClO_4_)_2_ (**6**) (blue) containing only (*R*,*R*)-enantiomers of the ligands.

In this work, a single-crystal structure was obtained for the complex [Fe^II^(±CDA-BQA)](CF_3_SO_3_)_2_ (**5**). The complex was prepared with the racemic version of ligand CDA-BQA*. In the CSD database, there are 29 structures of Fe(II) complexes of these types of ligands, with four pyridyl or quinoline groups connected by an ethylenediamine or cyclohexanediamine linker [51,52]. The only reported Fe(II) complex with a cyclohexanediamine linker is [Fe^II^(CDA-BPA*)](ClO_4_)_2_ (**6**) (CSD refcode YAMXAL) [41] The geometry of the newly synthesised [Fe^II^(CDA-BQA*)](CF_3_SO_3_)_2_ (**5**) (Figure 1) and [Fe^II^(CDA-BPA*)](ClO_4_)_2_ (**6**) is compared in Figure 2 and Table 1. Both complexes are prepared with racemic ligands, however, 5 crystallised as a racemate, while 6 has spontaneously resolved into its optical isomers, containing only the (*R,R*) enantiomer. While complex **6** has a regular octahedral geometry, the Fe-N bonds in **5** are elongated, forming a pentagonal bipyramidal geometry with an equatorial vacancy, as determined using the program FindGeo [53]. The reason for this is likely the steric crowding of the quinoline groups in **5**. The significantly longer Fe-N bond lengths (>2.2 Å) are in agreement with a high spin Fe(II) centre in **5**. The UV-Vis spectrum of **5** in acetonitrile is dominated by the intense π-π* band at 307 nm (=12,800 M^−1^ cm^−1^), and an additional broad feature of low intensity between 330 and 460 nm (λ_max_ = 356 with ε = 2080 M^−1^ cm^−1^) can be assigned to an MLCT transition. The weak intensity is consistent with the high spin state of the iron(II) centre [46].

### 2.2. Catalytic Oxidation Reactions

**Figure 3:**
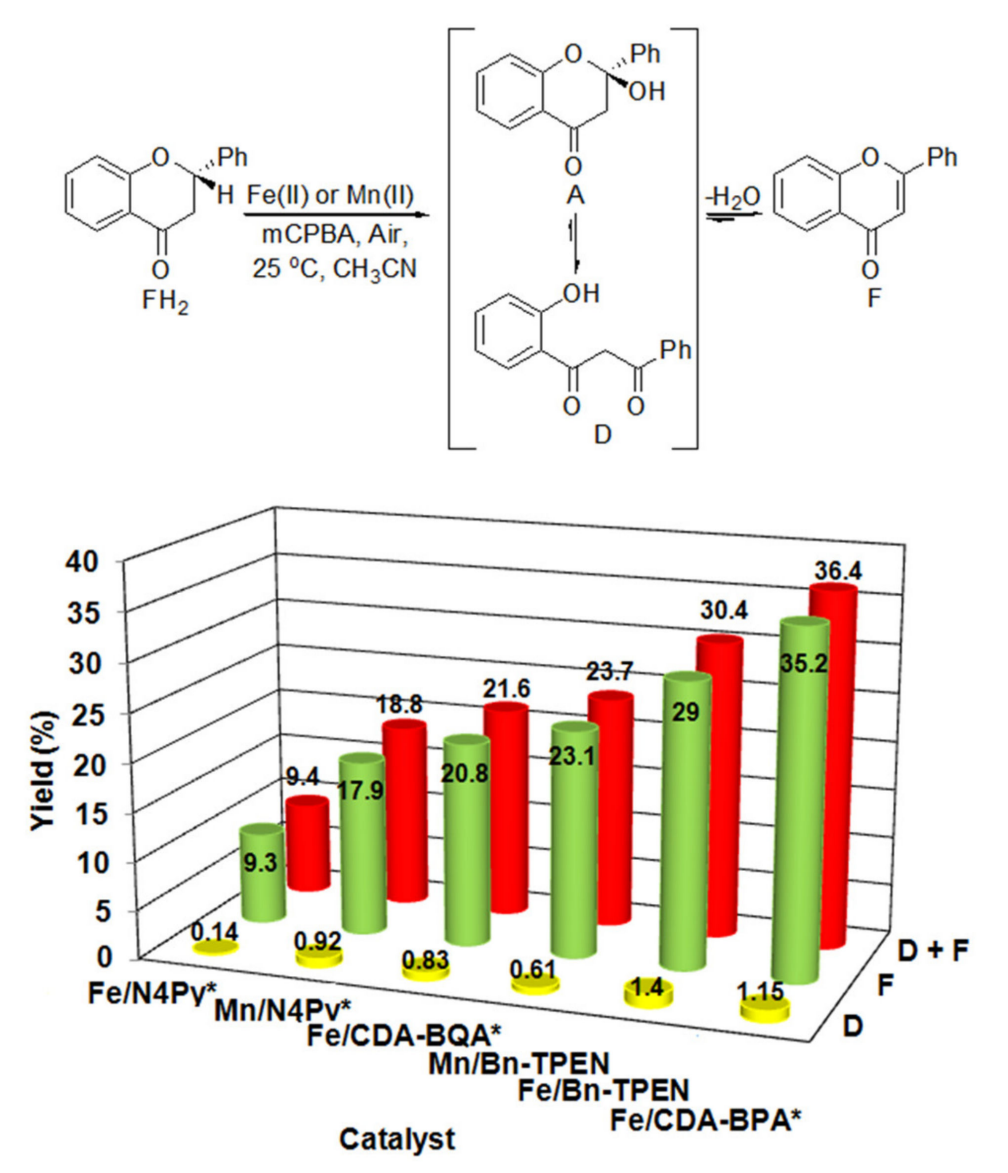
Comparison of the flavones-synthase activity of iron(II) and manganese(II) complexes. Conditions: [M^II^] = 5 mM, [FH_2_] = 100 mM and [*m*CPBA] = 500 mM in CH_3_CN at 25 °C.

The catalytic activities of the three ferrous and two manganese complexes [Fe^II^(Bn-TPEN)(CH_3_CN)]^2+^ (**3**), [Fe^II^(CDA-BQA*)]^2+^ (**5**), [Fe^II^(CDA-BPA*)]^2+^ (**6**), [Mn^II^(N4Py*)(CH_3_CN)]^2+^ (**2**) and [Mn^II^(Bn-TPEN)(CH_3_CN)]^2+^ (**4**) were studied in the oxidation of flavanone, utilising mCPBA (meta-chloroperoxybenzoic acid) as the co-oxidant. The oxidation reactions were carried out under standard catalytic conditions (5:100:500 ratio for the catalyst:substrate:oxidant) in acetonitrile at room temperature (Table 2 and Figure 3). Similarly to our previous results, it took less than 10 min to have about 30–36% and 19–24% yields (based on the substrate) for the iron(II)- and manganese(II)-catalysed reactions, respectively. Much lower yields (0.36 and 2.8%) were observed for the Fe(ClO_4_)_2_ and Mn(ClO_4_)_2_ salts, respectively. Both the iron(II) and manganese(II)-catalysed oxidations of flavanone produced flavones (F) as a major product in all cases in addition to two minor products, namely 2-hydroxy-2-phenyl-chroman-4-one (A, 2-hydroxy-flavanone) and its open tautomeric form 1-(2-hydroxy-phenyl)-3-phenyl-propane-1,3-dione (D). It was found earlier [46] that complex **1** together with mCPBA oxidizes flavanone, and a turnover number (TON) of 6.93 for F, 0.11 for D and 0.02 for A was obtained with an overall yield of 9.42%. Its manganese analogue produced an overall yield of 18.8%, TON for F = 3.58, TON for D = 0.19 and TON for A = ~0.01.

**Figure 4:**
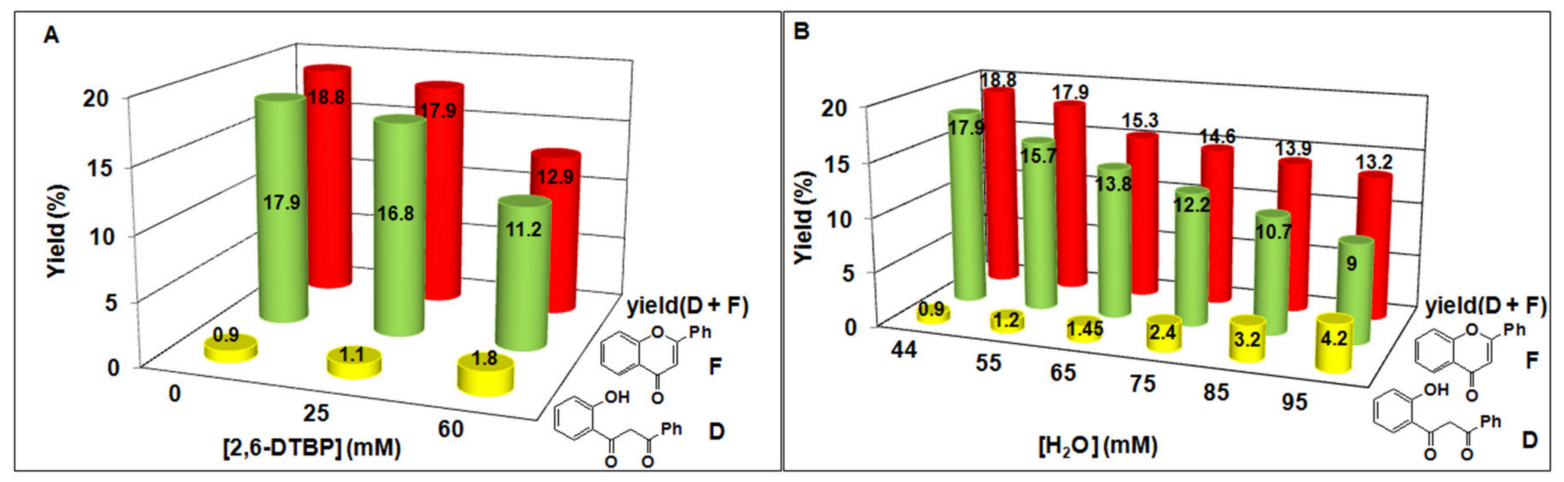
(**A**) Comparison of the product formation in the [Mn^II^(N4Py*)(CH_3_CN)]^2+^ (**2**) catalysed oxidation of flavanone with mCPBA as co-oxidant and 2,6-DTBP as a radical trapping agent. (**B**) Comparison of the product formation in the presence of added water.

It is important to mention that the amount of 1,3-dione (D) can be increased from 0.92% (TON = 0.19) to 4.2% (TON = 0.92) in the presence of H_2_O, suggesting an equilibrium step during the flavone formation (Figure 4B). However, when 2,6-di-tert-butylphenol (DTBP) was added significantly less flavanone was converted, suggesting a free-radical type mechanism as a parallel process of the metal-based oxidation (Figure 4A). Significantly higher yields were observed for **3** (30.4%, TON for F = 5.80, TON for D = 0.28), **5** (21.6%, TON for F = 4.16, TON for D = 0.17), **6** (36.35%, TON for F = 7.04, TON for D = 0.23) and **4**, suggesting clearly that the ligand framework and the nature of the metal influenced the catalytic activities of these complexes. The relative reactivities of iron(II) and manganese(II) complexes are in the order of [Fe^II^(CDA-BPA*)]^2+^ (**6**) > [Fe^II^(Bn-TPEN)(CH_3_CN)]^2+^ (**3**) > [Mn^II^(Bn-TPEN)(CH_3_CN)]^2+^ (**4**) > [Fe^II^(CDA-BQA*)]^2+^ (**5**) > [Mn^II^(N4Py*)(CH_3_CN)]^2+^ (**2**) > [Fe^II^(N4Py*)(CH_3_CN)]^2+^ (**1**). It should be underlined that the presence of both the theoretical tautomers (A and D) of the acid-labile intermediate of flavone strongly supports the hypothesis that flavone biosynthesis proceeds via 2-hydroxylation of flavanone. A similar mechanism was proposed for the cytochrome P450 monooxygenase (FS II) and other 2-oxoglutarate-dependent dioxygenases.

**Figure 5:**
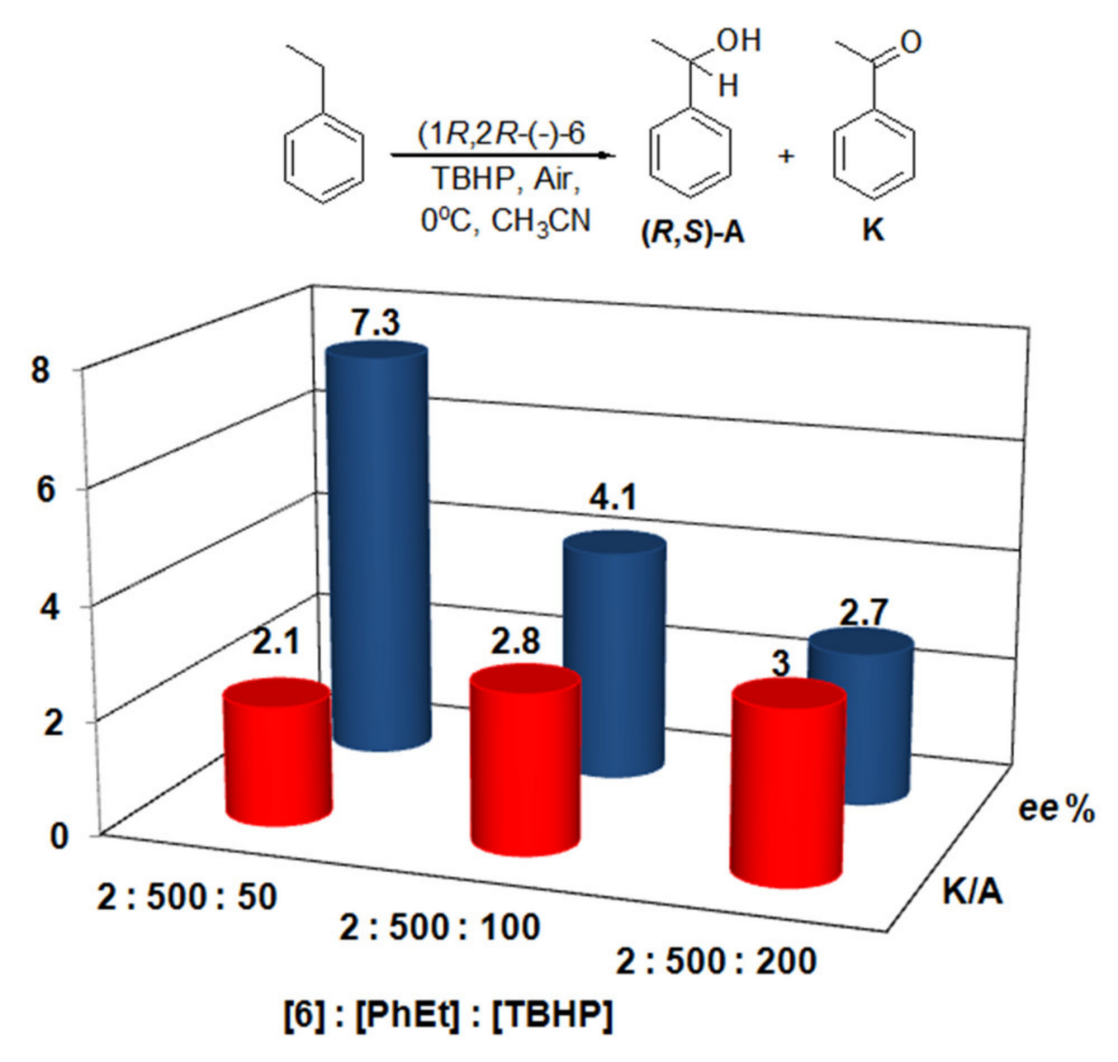
Dependence of the K/A ratio and the enantiomeric excess (ee%) on the oxidant concentration for the [Fe^II^(CDA-BPA*)]^2+^ (**6**) catalysed oxidation of ethylbenzene with TBHP in CH_3_CN at 0 °C.

Recently, chiral N4Py-type and L-proline derived aminopyridine containing oxoiron(IV) complexes were reported which could perform enantioselective oxidation of various substrates such as thioanisole, hydrocarbons, alkenes and substituted cyclohexanones [31,33]. To the best of our knowledge, these are the first examples of chiral nonheme oxoiron(IV) complexes tested in asymmetric C-H hydroxylation reactions. Since flavanone is a chiral molecule including benzylic C-H bonds, oxidative kinetic resolution (OKR) of racemic flavanones can in principle also be performed with a chiral iron catalyst and oxoiron(IV) intermediates. The oxidation of ethylbenzene that can be used as a model compound of flavanone, by chiral [Fe^IV^(O)(N4Py*)]^2+^ (**7**) species showed moderate enantioselectivities up to 33% ee as a result of a non-rebound mechanism including the epimerization of the long-lived alkyl radical before the rebound step [36]. To increase the enantioselectivity, we also examined the enantioselectivity of the [Fe^II^(CDA-BPA*)]^2+^ (**6**) catalyst and the in situ generated [Fe^IV^(O)(CDA-BPA^*^)]^2+^ (**11**) intermediate from **6** and PhIO in the asymmetric C-H hydroxylation of ethylbenzene utilizing TBHP and PhIO as co-oxidants (Figure 5 and Table 3). In these probe reactions, moderate enantioselective hydroxylation could be obtained in all cases, so the studies on oxidative kinetic resolution of racemic flavanones were discarded.

### 2.3. Stoichiometric Oxidation Reactions

We have previously reported for a family of aminopyridine containing oxoiron(IV) complexes, Fe^IV^(O)(N4Py) [N4Py = *N*,*N*’-bis(2-pyridylmethyl)-*N*-bis(2-pyridyl)methylamine], Fe^IV^(O)(TPA) [TPA = tris(2-pyridylmethyl)amine] that the reactivity in HAT and OAT reactions can be significantly enhanced by the introduction of 6-substituents on the pyridine rings (TPA), or by the replacement of one or more pyridyl arms of TPA, N4Py or N4Py* with quinolyl arms, despite their thermal stabilities [46,54,55]. Modification of the N4Py ligand as described above resulted in the following reaction rate orders for the flavanone oxidation: [Fe^IV^(O)(N2Py2Q*)]^2+^ > [Fe^IV^(O)(N4Py)]^2+^ > [Fe^IV^(O)(N4Py*)]^2+^ (Table 4), where the most reactive complex reacts 80-fold faster than the slowest one [46]. The stability and reactivity of the complexes are also greatly influenced by the number of nitrogen-donor atoms and their hybrid state. For example the oxidation of ethylbenzene by pentadentate [Fe^IV^(O)(Bn-TPEN)]^2+^ (**9**) complex with two N(sp^3^) and 3 N(sp^2^) donor atoms react 20-fold faster than that of [Fe^IV^(O)(N4Py)]^2+^ [37]. Taking these results into account, further studies were performed using the previously published [Fe^IV^(O)(Bn-TPEN)]^2+^ (**9**) and the newly synthetised Fe^IV^(O)(CDA-BPA)]^2+^ (**11**) complexes for the stoichiometric oxidation of flavanone. Complexes **9** and **11** could be generated from their iron(II) precursor complexes by literature methods using 2 equiv solid PhIO for 30–40 min [37,38]. The reaction of **6** with PhIO in acetonitrile at room temperature results in a green species (**11**), characterised by a characteristic absorption band at 740 nm (ε ≈ 450 M^−^^1^ cm^−1^), which can be assigned as a ligand-field (d-d) transition on the low-spin (S = 1) Fe(IV) centre. This species is much less stable (t_1/2_ ≈ 2 h at 25 °C) than that was found for **9** (t_1/2_ ≈ 6 h at 25 °C). Their decay can be significantly enhanced by the addition of flavanone.

**Figure 6:**
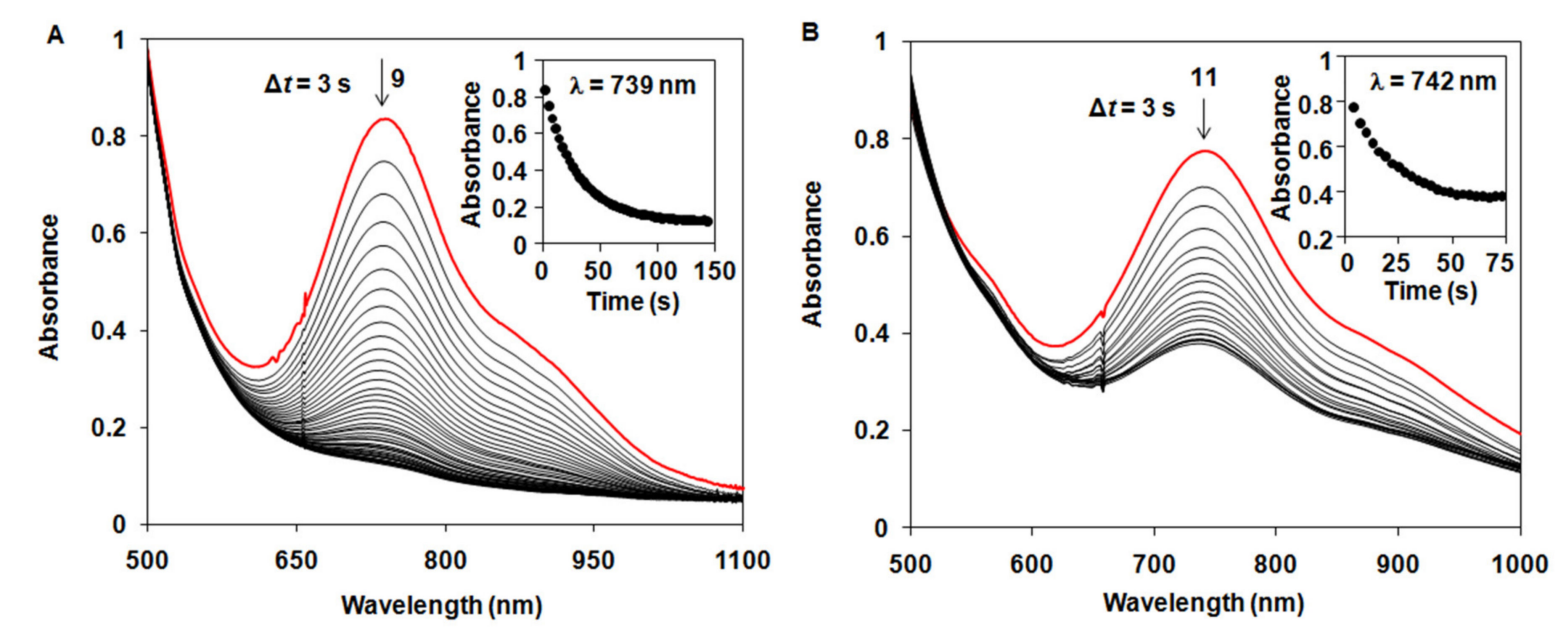
(**A**) UV-Vis spectral changes of **9** (2 mM, red line) upon addition of flavanone (50 mM) in CH_3_CN at 10 °C. The inset shows the time course of the decay of **9** monitored at 739 nm. (**B**) UV-Vis spectral changes of **11** (2 mM, red line) upon addition of flavanone (50 mM) in CH_3_CN at 10 °C. The inset shows the time course of the decay of **11** monitored at 742 nm.

**Figure 7:**
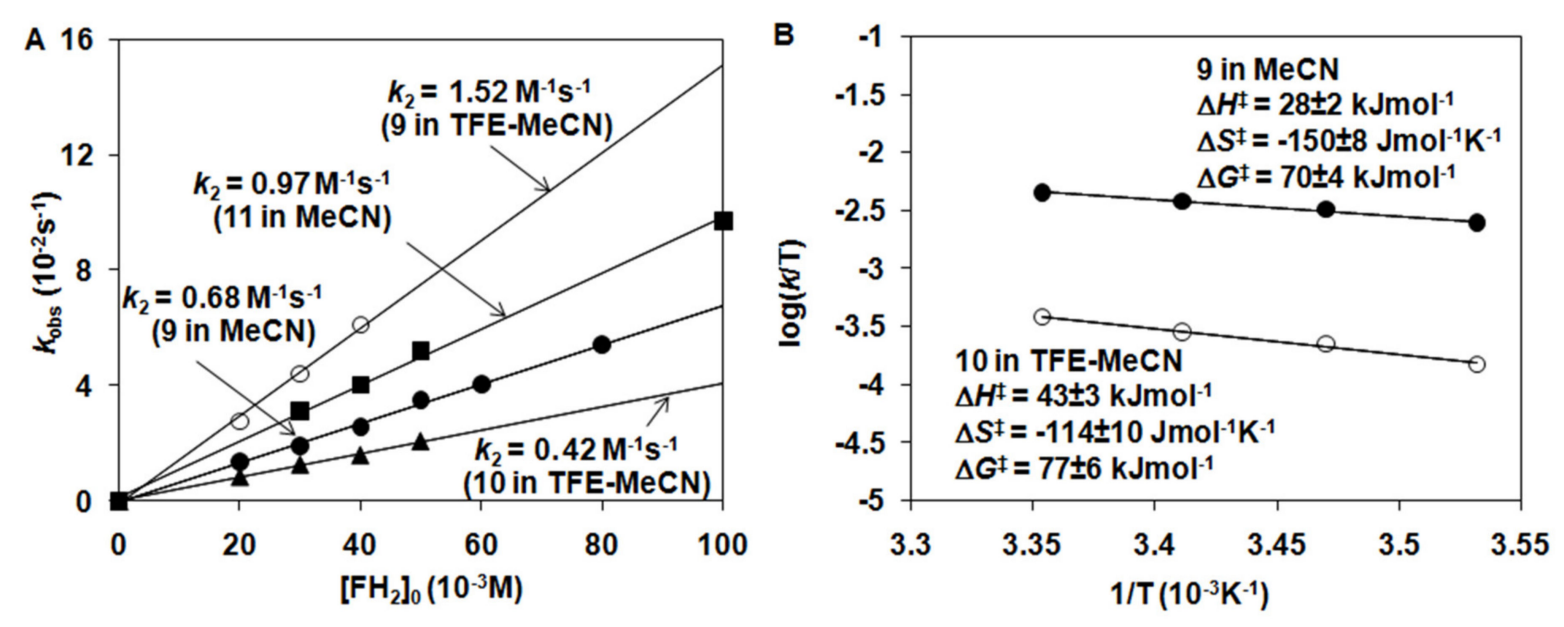
(**A**) Determination and comparison of second-order rate constants by plotting k_obs_ values against flavanone concentration for a series of M^IV^(O) complexes in CH_3_CN at 10 °C, [M]_0_ = 2 mM. (**B**) Eyring plots of log k/T versus 1/T for **9** and **10**, [9,10] = 2 mM, [FH_2_] = 50 mM.

The detailed kinetic measurements were carried out in CH_3_CN and CH_3_CN/CF_3_CH_2_OH (TFE) = 1:1 solutions at 10–25 °C, and the decay of the oxoiron(IV) species **9** and **11** during the flavanone oxidation was followed as a decrease in absorbance at ~740 nm (Figure 6 and Tables S2–S5). The yields of flavone were around ~80% for both complexes. The reaction rates in the presence of 10–50 times excess of substrate obeyed pseudo-first-order kinetics, and the pseudo-first-order rate constants (k_obs_) were directly proportional to the concentration of flavanone, from which the reaction rate constants (k_2_) are 0.68 ± 0.027 M^−^^1^ s^−1^ and 0.97 ± 0.04 M^−^^1^ s^−1^ for **9** and **11** at 10 °C, respectively (Figure 7 and Table 4). These values are three orders of magnitude higher than those observed for the previously published [Fe^IV^(O)(N2Py2Q*)]^2+^ species [46], showing clearly that the ligand framework significantly influenced the reactivity of the oxoiron(IV) species.

**Figure 8:**
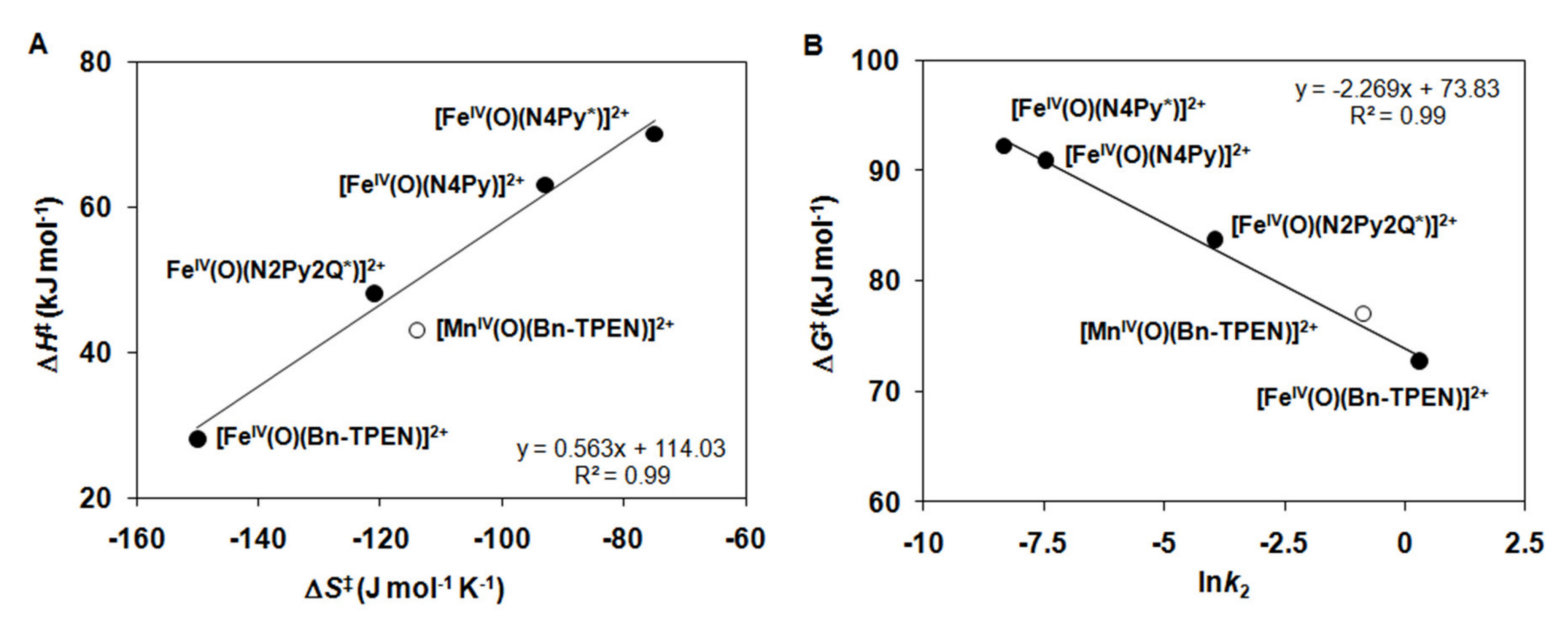
(**A**) Isokinetic plot and (**B**) plot of Δ*G*^‡^ versus lnk_2_ for the oxidation of flavanone with various oxomanganese(IV) and oxoiron(IV) complexes.

The relative reactivity of oxoiron(IV) complexes is in the order of [Fe^IV^(O)(CDA-BPA*)]^2+^ (**11**) > [Fe^IV^(O)(Bn-TPEN)]^2+^ (**9**) > [Fe^IV^(O)(N2Py2Q*)]^2+^ > [Fe^IV^(O)(N4Py)]^2+^ > [Fe^IV^(O)(N4Py*)]^2+^ (**7**), which is consistent with our catalytic results. Based on the temperature dependence of the reactivity of **9** (with Δ*H*^‡^ = 28 ± 2 kJ mol^−1^, Δ*S*^‡^ = −150 ± 8 J mol^−1^ K^−1^, Δ*G*^‡^ = 72.7 kJ mol^−1^), the value of -TΔ*S*^‡^ determined was bigger than Δ*H*^‡^, indicating an entropy-controlled reaction, contrary to the previously reported enthalpy-controlled reactions with N4Py-type ligands. As a result of a compensation effect increasing activation, enthalpies are offset by increasingly positive entropies yielding Δ*H*^‡^ = 114 kJ mol^−1^ at the intercept (Figure 8A). The experimentally determined difference between Δ*G*^‡^ values is 20 kJ mol^−1^, which is significant and consistent with the observed reaction rate order (Figure 8B).

**Figure 9:**
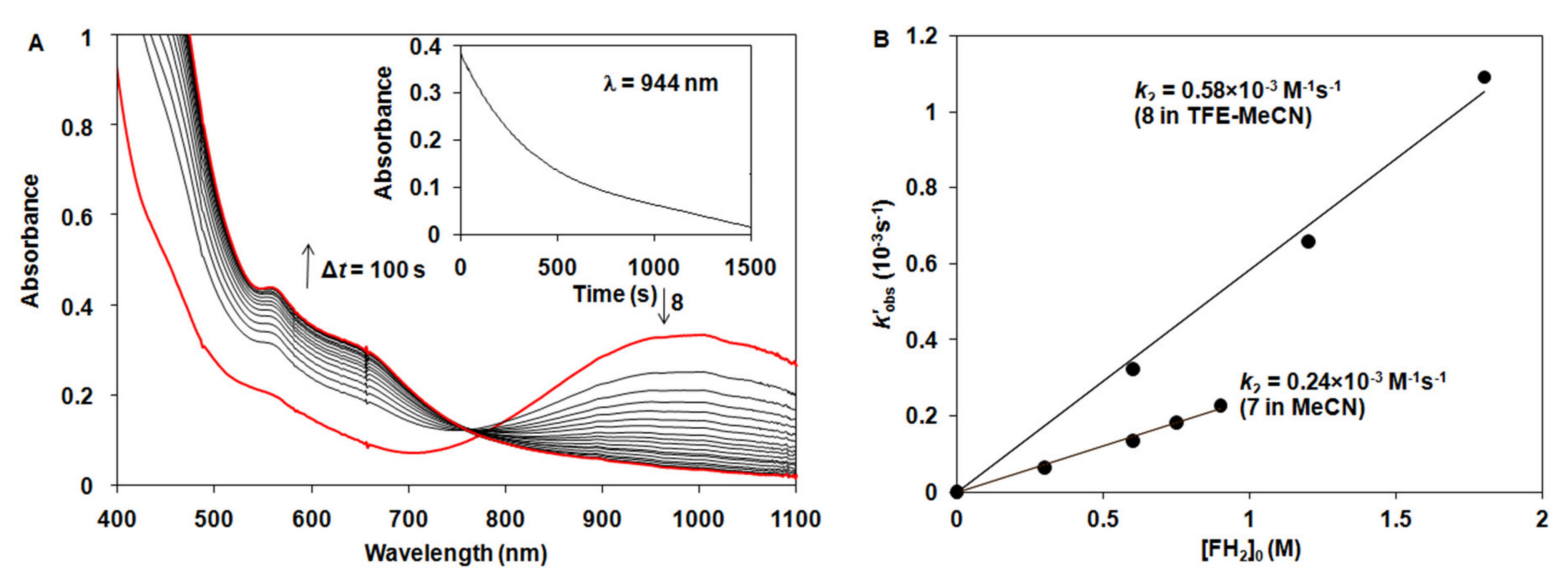
(**A**) UV-Vis spectral changes of **8** (2 mM, red line) upon addition of flavanone (1.8 M) in CH_3_CN/TFE at 25 °C. The inset shows the time nd course of the decay of **8** monitored at 944 nm. (**B**) Determination and comparison of second-order rate constants by plotting k_obs_ values against flavanone concentration for complexes **7** and **8** in CH_3_CN and CH_3_CN/TFE at 25 °C, [M]_0_ = 2 mM.

To elucidate the role of the metal cofactor, such manganese and iron containing systems have been chosen from the literature where the structure of the high valent metal oxo intermediates are already known. Our choice fell on the [Mn^IV^(O)(N4Py*)]^2+^ (**8**) and [Mn^IV^(O)(Bn-TPEN)]^2+^ (**10**) complexes. These intermediates can be generated in TFE and TFE/CH_3_CN by the use of PhIO as an oxidant, and the oxidation of flavanone can be investigated following their decrease in absorbances at 944 nm (**8**) and 1040 nm (**10**), respectively (Figure 9A).

**Scheme 3:**
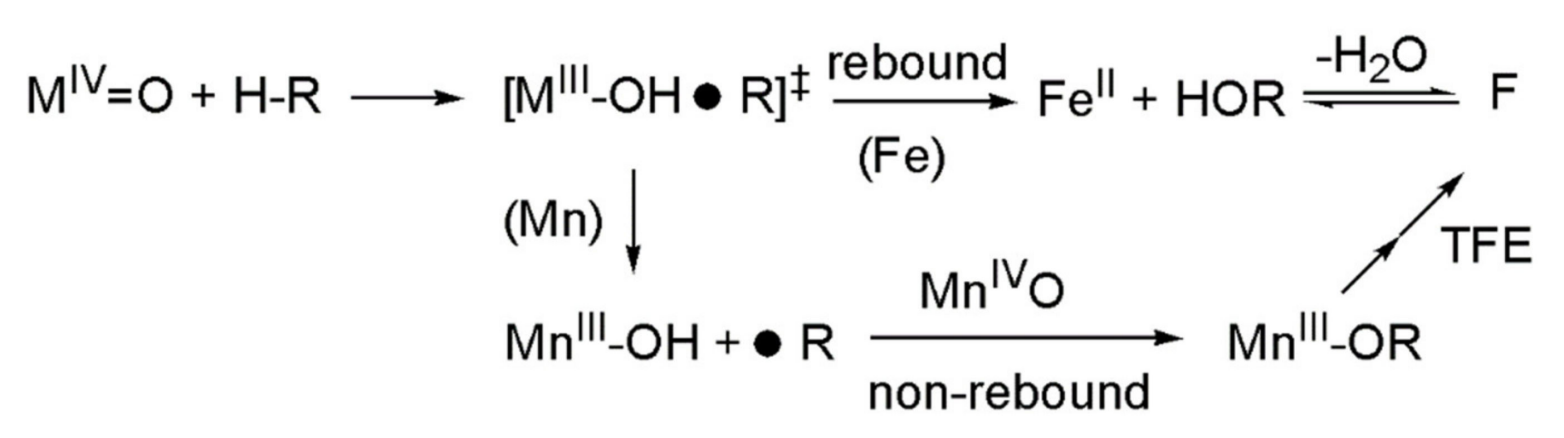
Proposed mechanism for the C-H activation by oxoiron and oxomanganese complexes.

Due to the correct comparison of the iron and manganese-containing system, we first investigated the effect of the solvent on the reaction rate. The reaction of [Fe^IV^(O)(Bn-TPEN)]^2+^ (**9**) with flavanone in CH_3_CN/TFE (1:1) resulted in a 2-fold increase in rate, which is not considered significant. Considering the solvent effect, almost the same reaction rate was observed for the [Fe^IV^(O)(N4Py*)]^2+^ (**7**) and [Mn^IV^(O)(N4Py*)]^2+^ (**8**) complexes with k_2_ = 0.24(1) × 10^−3^ M^−1^ s^−1^ and k_2_ = 0.58(3) × 10^−3^ M^−1^ s^−1^ at 25 °C, respectively (Figure 9B). Comparing the reactions of [Fe^IV^(O)(Bn-TPEN)]^2+^ (**9**) and [Mn^IV^(O)(Bn-TPEN)]^2+^ (**10**) under the same conditions, a 3.5-fold difference in reaction rate was observed in favour of iron (Table 4 and Figure 7)). The difference in reaction rates and yields of products (~80% flavone for **9** and ~40% flavone for **10** based on the complex concentration) can be explained by a different mechanism based on the literature data. While in the case of oxoiron(IV) complexes the reactions occur mostly via an oxygen-rebound mechanism [56], in the case of manganese the process involving C-H activation can be described mainly by a non-rebound mechanism with a smaller reaction rate (Scheme 3) [40].

## 3. Materials and Methods

Reactions were carried out in ordinary glassware and chemicals were used as purchased from commercial suppliers without further purification. GC analyses were performed on an Agilent 6850 gas chromatograph equipped with a flame ionization detector and a 30 m SUPELCO BETA DEX225 (CHIRASIL-L-VAL) (Sigma-Aldrich, Budapest, Hungary) column. ESI-MS samples were analysed using a triple quadruple Micromass Quattro spectrometer (Waters, Milford, MA, USA) and an HPLC-MS system (Agilent Technologies 1200, Budapest, Hungary) coupled with a 6410 Triple-Quadrupole mass spectrometer, operating in a positive ESI mode. Synthesis of the ligand was carried out in a microwave reactor (CEM Discover), (CEM Inc, Scottsdale, AZ, USA) monitored by TLC on aluminium oxide 60 F_254_ neutral plates and detected with a UV lamp (254 nm). NMR spectra were obtained on a Bruker Avance 300 (Bruker Biospin AG, Fällanden, Switzerland) or 600 spectrometers, operating at 300 or 600 MHz for ^1^H and 75 or 150 MHz for ^13^C. The spectra are recorded at room temperature. Chemical shifts, *δ* (ppm), indicate a downfield shift from the residual solvent signal (*δ_H_*: 1.94 ppm, *δ_C_*: 118.26 ppm for CD_3_CN and *δ_H_*: 7.26 ppm, *δ_C_*: 77.16 ppm for CDCl_3_). Coupling constants, *J*, are given in Hz. The syntheses of most of the complexes used in this study have been previously reported: these complexes and the corresponding references are listed as follows: [Fe^II^(Bn-TPEN)(CH_3_CN)]^2+^ (**3**), [Fe^IV^(O)(Bn-TPEN)]^2+^ (**9**) [37,38], [Mn^II^(Bn-TPEN)(CH_3_CN)]^2+^ (**4**), [Mn^IV^(O)(Bn-TPEN)]^2+^ (**10**) [40], [Mn^II^(N4Py*)(CH_3_CN)]^2+^ (**2**), [Mn^IV^(O)(N4Py*)]^2+^ (**8**) [39], [Fe^II^(CDA-BPA*)]^2+^ (**6**) [41].

Synthesis of ligands CDA-BPA* and CDA-BQA*. The synthesis was performed according to a modified previously reported procedure [47]. The amine (1 eq.), K_2_CO_3_ (12 eq.), 2-(chloromethyl)pyridine hydrochloride or 2-(chloromethyl)quinoline hydrochloride (4 eq.) and KI (1 eq.) were suspended in 50 mL of acetonitrile. The reaction mixture was heated in a microwave reactor (50 W, reflux) for 1 h. The solvent was evaporated in a vacuum, the residue suspended in ethyl acetate and washed three times with brine and saturated NaHCO_3_, the organic layer dried over anhydrous sodium sulphate, filtered and evaporated in a vacuum. The crude ligand was purified by automated flash chromatography on a pre-packed neutral alumina column (48 g).

±CDA-BPA: (±)-*trans*-1,2-Diaminocyclohexane (120.0 μL, 1.0 mmol), K_2_CO_3_ (1658.4 mg, 12.0 mmol), 2-(chloromethyl)pyridine hydrochloride (656.2 mg, 4.0 mmol), KI (166.0 mg, 1.0 mmol). Automated flash chromatography 0→5% dichloromethane in methanol (*R*_f_ = 0.42, 5% dichloromethane in methanol). Yield: 303.0 mg (0.6 mmol, 63%), brown oil.

(*R,R*)-CDA-BPA: (1*R*,2*R*)-(−)-1,2-Diaminocyclohexane (114.2 mg, 1.0 mmol), K_2_CO_3_ (1658.4 mg, 12.0 mmol), 2-(chloromethyl)pyridine hydrochloride (656.2 mg, 4.0 mmol), KI (166.0 mg, 1.0 mmol). Automated flash chromatography 0→5% dichloromethane in methanol. Yield: 337.5 mg (0.7 mmol, 71%), light brown powder. ^1^H NMR (300 MHz, CD_3_CN) *δ*: 8.39 (d, *J* = 4.7 Hz, 4H), 7.61 (d, *J* = 7.8 Hz, 4H), 7.55–7.41 (m, 4H), 7.18–7.03 (m, 4H), 3.70 (d, *J* = 14.3 Hz, 4H), 3.58 (d, *J* = 14.3 Hz, 4H), 2.85–2.66 (m, 2H), 1.79–1.61 (m, 2H), 1.36–1.23 (m, 1H), 1.23–1.00 (m, 4H), 0.96–0.79 (m, 1H). ^13^C NMR (75 MHz, CD_3_CN) *δ*: 161.6, 149.5, 136.7, 124.5, 122.6, 60.8, 56.5, 26.8, 25.7. ESI-MS (*m*/*z*): 479.2 (M + H^+^, 100%).

±CDA-BQA: (±)-*trans*-1,2-Diaminocyclohexane (70.5 μL, 0.6 mmol), K_2_CO_3_ (970.2 mg, 7.0 mmol), 2-(chloromethyl)quinoline hydrochloride (500.0 mg, 2.3 mmol), KI (96.3 mg, 0.6 mmol). No further purification was required, (*R*_f_ = 0.28, ethyl acetate:hexane 1:1). Yield: 372.3 mg (0.5 mmol, 94%), yellow powder.

(*R*,*R*)-CDA-BQA: (1*R*,2*R*)-(−)-1,2-Diaminocyclohexane (44.5 mg, 0.4 mmol), K_2_CO_3_ (651.6 mg, 4.7 mmol), 2-(chloromethyl)quinoline hydrochloride (378.9 mg, 1.6 mmol), KI (64.7 mg, 0.4 mmol). Automated flash chromatography 10→40% ethyl acetate in hexane. Yield: 202.3 mg (0.3 mmol, 76%), light yellow powder. ^1^H NMR (300 MHz, CDCl_3_) *δ*: 7.99 (d, *J* = 8.3 Hz, 4H), 7.91 (d, *J* = 8.5 Hz, 4H), 7.77 (d, *J* = 8.5 Hz, 4H), 7.72–7.59 (m, 8H), 7.52–7.41 (m, 4H), 4.01 (d, *J* = 14.1 Hz, 4H), 3.85 (d, *J* = 14.1 Hz, 4H), 3.00–2.81 (m, 2H), 2.49–2.27 (m, 2H), 1.88–1.69 (m, 2H), 1.25–0.94 (m, 4H). ^13^C NMR (75 MHz, CDCl_3_) *δ*: 161.3, 147.7, 135.8, 129.3, 129.1, 127.6, 127.4, 126.1, 122.2, 60.7, 56.6, 29.8, 26.0, 24.4. ESI-MS (*m*/*z*): 679.4 (M + H^+^, 100%).

Fe^II^(CDA-BQA*) (**5**): Ligand ± CDA-BQA (14.2 mg, 0.02 mmol) and Fe(CF_3_SO_3_)_2_ (7.4 mg, 0.02 mmol) were dissolved in dry acetonitrile and mixed. The reaction vessel was placed in a tank with dry diethyl ether for vapour diffusion. The complex preparation was performed in a drybox in an atmosphere of argon. After 1 day, crystals suitable for single crystal X-ray diffraction were obtained. UV-Vis [MeCN = acetonitrile] [λ_max_, nm logε] = 203 (5.03), 231 (4.78), 307 (4.13). FT-IR bands (KBr pellet cm^−1^): 3429 m, 3068 w, 2933 w, 2859 w, 1701 s, 1653 s, 1600 w, 1279 s, 1166 s, 1029 s, 784 w, 667 m, 638 m, 522 w. Anal Calcd for C_48_H_42_F_6_FeN_6_O_6_S_2_: C, 55.82; H, 4.10; N, 8.14. Found: C, 55.79; H, 4.15; N, 8.19. X-ray: Chemical formula: C_50_H_45_F_6_FeN_7_O_6_S_2_, Formula weight: 1073.90, Crystal system: orthorhombic, Space group: *Cmca*, *a* (Å): 19.7874(9), *b* (Å): 42.5557(15), *c* (Å): 12.4013(4), Volume (Å^3^): 10442.7(7), *Z*: 8, Calc. density (g cm^−1^): 1.366, Temperature (K): 293(2), Abs. coeff. (mm^−1^): 3.715, *F* (0 0 0): 4432, Obs. reflections: 5532, Goodness-of-fit: 1.055, *R*_1_: 0.0761, *w*R_2_: 0.2287 (*w*=1⁄[*σ*^2^(*F*_o_^2^)+(*0.1721P*)^2^+*1.4205P*] and *P = (F_o_^2^* +2*F_c_*^2^)/3). CCDC deposit number 2082080 contains the supplementary crystallographic data for this paper. These data can be obtained free of charge via http://www.ccdc.cam.ac.uk/conts/retrieving.html (accessed on 30 April 2021) (or from the Cambridge Crystallographic Data Centre, 12, Union Road, Cambridge CB2 1EZ, UK; fax: +44 1223 336033) (Table S1).

Catalytic oxidation of ethylbenzene was carried out under thermostatic (273 K) conditions. In a typical reaction, 500 µL of TBHP (diluted from 70% TBHP) solution in CH_3_CN was delivered by a syringe pump in air to a stirred solution (1 mL) of **6** catalysts, and ethylbenzene inside a vial. The final concentrations were 2 mM catalyst **6**, 50 mM (100, 200 mM) oxidant, and 500 mM substrate. The mixture was stirred for 15 min. The products were identified by GC analysis, and their yields were determined by comparison with authentic compounds using bromobenzene (25.00 × 10*^−^*^3^ M) as an internal standard in the reactions.

Complex **6** (2.00 × 10*^−^*^3^ M) was dissolved in acetonitrile (2 mL), then iodosobenzene (16.00 × 10^−3^ M) and ethylbenzene (1500 × 10^−3^ M) were added to the solution in CH_3_CN at 273 K. The mixture was stirred for 3 h and the products were identified by GC analysis, and their yields were determined by comparison with authentic compounds using bromobenzene (4.00 × 10*^−^*^3^ M) as an internal standard in the reactions. Enantiomeric excess was determined with GC analysis on a chiral column: (*ee* = [R] − [S])/([R] + [S]).

Catalytic oxidation of flavanone was carried out under thermostatic (298 K) conditions. In a typical reaction, 5.8 mg **2** (5 mM) and 33.6 mg flavanone (100 mM) was dissolved in 1 mL CH_3_CN, and 169 mg mCPBA (500 mM) in 500 µL CH_3_CN was delivered by syringe pump to a stirred solution. After syringe pump addition (5 min the solution was stirred for 5 min and a known amount of PhBr (0.315 mmol) was added as an internal standard. The iron complex was removed by passing the reaction mixture through a silica column followed by elution with ethyl acetate. The products (1,3-dione (D) and flavone) were identified by GC/MS and confirmed by comparison with authentic samples. Flavone is commercially available and it was purchased from Sigma-Aldrich. 1-(2-hydroxy-phenyl)-3-phenyl-propane-1,3-dione (D): o-Benzoyloxyacetophenone has been prepared by the action of benzoyl chloride on a pyridine solution of o-hydroxyacetophenone. Its rearrangement to 1-(2-hydroxy-phenyl)-3-phenyl-propane-1,3-dione (D) by alkali has been described [57]. (F) *m*/*z*: 222 (85.92%), 194 (58.9%), 165 (18%) 120 (100%), 92 (95,2%). 63 (35.3%). 1,3-dione (D): *m*/*z*: 240 (15.1%), 223 (8.3%), 121 (25.2%), 120 (7.3%), 106 (7.2%), 105 (100%), 77 (30%), 69 (6.0%), 65 (9.3%), 51 (4.5%), 39 (8.3%).

Stoichiometric reactions were carried out under thermostatic conditions at 10 °C in 1 cm quartz cuvettes. In a typical experiment, [Fe^II^(Bn-TPEN)(CH_3_CN)]^2+^ (2 × 10^−3^ M) was dissolved in acetonitrile(CH_3_CN-TFE) (2.0 cm^3^), then iodosobenzene (4 × 10^−3^ mM) was added to the solution. The mixture was stirred for 50 min then excess iodosobenzene was removed by filtration. Flavanone (50 mM) was added to the solution and the reaction was monitored by UV-Vis spectrophotometer (Agilent 8453) at 739 nm (ε = 450 M^−^^1^ cm^−1^).

## 4. Conclusions

In conclusion, we previously found that N4Py-based iron(II) complexes capable of carrying out 2,3-desaturation of flavanone via 2-hydroxyflavanone intermediate formation can act as a functional flavone synthase model. As a continuity of this study, efforts have been made to enhance the catalytic activity by the use of TPEN-type ligands and investigate the role and effect of the metal cofactor through manganese and iron complexes with the same ligand framework. Comparing the reactions of [Fe^IV^(O)(Bn-TPEN)]^2+^ (**9**) and [Mn^IV^(O)(Bn-TPEN)]^2+^ (**10**) towards flavanone under the same conditions, a 3.5-fold difference in reaction rate was observed in favour of iron, and this value is three orders of magnitude higher than that was observed for the previously published [Fe^IV^(O)(N2Py2Q*)]^2+^ species. The relative reactivity of oxoiron(IV) complexes is in the order of [Fe^IV^(O)(CDA-BPA*)]^2+^ (**11**) > [Fe^IV^(O)(Bn-TPEN)]^2+^ (**9**) > [Fe^IV^(O)(N2Py2Q*)]^2+^ > [Fe^IV^(O)(N4Py)]^2+^ > [Fe^IV^(O)(N4Py*)]^2+^ (**7**), which is consistent with our catalytic results, and shows that addition of cyclohexanediamine as the chiral element successfully lead to increase in the catalytic activity. Although we have reported the first example of efficient flavanone oxidation by iron and manganese complexes, detailed studies are underway to elucidate the mechanisms of flavone formation.

## Figures and Tables

**Table 1:** Selected bond lengths of complex [Fe^II^(±CDA-BQA*)](CF_3_SO_3_)_2_ (**5**) compared to the literature example of [Fe^II^(CDA-BPA*)](ClO_4_)_2_ (**6**) complex. The geometry was determined using the program FindGeo [53].

	Fe-N1/Å	Fe-N2/Å	Fe-N3/Å	Geometry/RMSD/Å
[Fe^II^(±CDA-BQA*)](CF_3_SO_3_)_2_ (**5**)	2.194	2.238	2.252	Pentagonal bipyramid with a vacancy (equatorial) (regular) 0.271
[Fe^II^(CDA-BPA*)](ClO_4_)_2_ (**6**) [41]	1.993 2.003	2.012 2.038	1.994 1.993	Octahedron (regular) 0.369

**Table 2:** Catalytic oxidation of flavanone (FH_2_) carried out by **2**, **3**, **4**, **5** and **6** with mCPBA in CH_3_CN at 25 °C.

M^II^	[H_2_O] /mM	[M^II^] /mM	[FH_2_] /mM	[*m*CPBA] /mM	Yield (F) /%	TON	Yield (D) ^c^ /%	TON	Yield_t_ /%
[Fe^II^(CDA-BPA*)]^2+^ (**6**)	44	5	100	500	35.2	7.04	1.15	0.23	36.35
[Fe^II^(Bn-TPEN)]^2+^ (**3**)	44	5	100	500	29	5.8	1.4	0.28	30.4
[Fe^II^(CDA-BQA*)]^2+^ (**5**)	44	5	100	500	20.8	4.16	0.83	0.17	21.63
[Mn^II^(N4Py*)]^2+^ (**2**)	44	5	100	500	17.9	3.58	0.92	0.19	18.8
[Mn^II^(N4Py*)]^2+^ (**2**)	55	5	100	500	15.7	3.14	1.2	0.23	16.9
[Mn^II^(N4Py*)]^2+^ (**2**)	65	5	100	500	13.8	2.76	1.45	0.3	15.25
[Mn^II^(N4Py*)]^2+^ (**2**)	75	5	100	500	12.2	2.44	2.4	0.48	14.64
[Mn^II^(N4Py*)]^2+^ (**2**)	85	5	100	500	10.7	2.14	3.2	0.64	13.9
[Mn^II^(N4Py*)]^2+^ (**2**)	95	5	100	500	9	1.88	4.2	0.92	13.2
[Mn^II^(N4Py*)]^2+^ (**2**)	44	5	100 ^a^	500	16.8 ^a^	3.36	1.1	0.2	17.9
[Mn^II^(N4Py*)]^2+^ (**2**)	44	5	100 ^b^	500	11.2 ^b^	2.24	1.8	0.36	12.95
[Mn^II^(Bn-TPEN)]^2+^ (**4**)	44	5	100	500	23.1	4.62	0.61	0.12	23.7

^a^ In the presence of 2,6-di-*tert*-butylphenol (2,6-DTBP), [2,6-DTBP] = 25 mM. ^b^ In the presence of 2,6-di-*tert*-butylphenol (2,6-DTBP), [2,6-DTBP] = 60 mM.^c^ Yield of A is less than 0.1%.

**Table 3:** Catalytic oxidation of ethylbenzene carried out by (*R,R*)-(−)-**6** and (*R,R*)-(−)-**5** with various co-oxidants ^a^.

[6]:[PhEt]:[Co-oxidant]	TON (A) ^b^	TON (K) ^c^	Yield /% ^d^	K/A	*ee* /%
2:500:50 (TBHP)	1.38	2.83	16.84	2.05	7.25 (R)
2:500:100 (TBHP)	1.82	5.20	14.04	2.86	4.13 (R)
2:500:200 (TBHP)	2.06	6.17	8.23	3.00	2.68 (R)
2:1500:16 (PhIO)	0.96	2.14	38.75	2.23	12.21 (R)
[5]:[PhEt]:[Co-oxidant]	TON (A ^b^)	TON (K ^c^)	Yield /% ^d^	K/A	*ee* /%
2:500:100 (TBHP)	-	10.08	20.16	-	-

^a^ Reaction conditions: see Experimental section. ^b^ 1-Phenylethanol. ^c^ Acetophenone. ^d^ Based on oxidant.

**Table 4:** Rate constants and activation parameters for the oxidation of flavanone with oxometal(IV) complexes.

Complex	10^3^*k*_2_ /s^−1^ M^−^^1^	Δ*H*^‡^ /kJ mol^−1^	Δ*S*^‡^ /J mol^−1^ K^−1^	Δ*G*^‡^ /kJ mol^−1^	Solvent /T	Refs.
[Fe^IV^(O)(N4Py*)]^2+^ (**7**)	0.24 ± 0.01	70 ± 3	−75 ± 9	92.2	CH_3_CN (25 °C)	[46]
[Fe^IV^(O)(N4Py)]^2+^	0.57 ± 0.03	63 ± 4	−93 ± 13	90.9	CH_3_CN (25 °C)	[46]
[Fe^IV^(O)(N2Py2Q*)]^2+^	19.2 ± 1.1	48 ± 2	−121 ± 8	83.7	CH_3_CN (25 °C)	[46]
[Fe^IV^(O)(N2Py2Q*)]^2+^	6.50 ± 0.32				CH_3_CN (10 °C)	[46]
[Fe^IV^(O)(Bn-TPEN)]^2+^ (**9**)	1340 ± 67	28 ± 2	−150 ± 8	72.7	CH_3_CN (25 °C)	This work
[Fe^IV^(O)(Bn-TPEN)]^2+^ (**9**)	680 ± 27				CH_3_CN (10 °C)	This work
[Fe^IV^(O)(Bn-TPEN)]^2+^ (**9**)	1520 ± 90				CH_3_CN/TFE (10 °C)	This work
[Fe^IV^(O)(CDA-BPA*)]^2+^ (**11**)	970 ± 40				CH_3_CN (10 °C)	This work
[Mn^IV^(O)(N4Py*)]^2+^ (**8**)	0.58 ± 0.03				CH_3_CN/TFE (25 °C)	This work
[Mn^IV^(O)(Bn-TPEN)]^2+^ (**10**)	420 ± 15	43 ± 3	−114 ± 10	76.7	CH_3_CN/TFE (10 °C)	This work

## Data Availability

Not available.

## Supplementary Materials

The following are available online. Figure S1: Ligands **1a** (±cda-bpa)–**2b** (±cda-bqa); Figure S2: Packing of [Fe^II^(CDA-BQA*)]^2+^ complexes in rac-5. Individual enantiomers are shown in red and blue colours, respectively. Triflate anions and acetonitrile molecules are omitted for clarity; Table S1: Experimental data for the X-ray diffraction studies; Table S2: The calculated *k_obs_* values in the reaction of **9** and flavanone in MeCN; Table S3: The calculated *k_obs_* values in the reaction of **9** and flavanone in MeCN/TFE; Table S4: The calculated *k_obs_* values in the reaction of **11** and flavanone in MeCN; Table S5: The calculated *k_obs_* values in the reaction of **10** and flavanone in MeCN/TFE.
