# Supplementary material for: Comparison of Nonheme Manganese- and Iron-Containing Flavone Synthase Mimics
Source: Molecules. 2021 May 27;26(11):3220. doi: 10.3390/molecules26113220 (PMC8198008; doi:10.3390/molecules26113220)
Supplement: Supplementary file 1 [file molecules-26-03220-s001.zip › molecules-1232702-supplementary.pdf]

Supporting Information

Comparison of Nonheme Manganese- and Iron-Containing Flavone Synthase Mimics

Dóra Lakk-Bogáth [1], Natalija Pantalon Juraj [2], Bashdar I. Meena [1], Berislav Perić, [2] Srećko I. Kirin [2] and József Kaizer [1,*]

1 *Research Group of Bioinorganic and Biocoordination Chemistry, University of Pannonia, H-8201 Veszprém, Hungary*

2 *Division of Materials Chemistry, Ruđer Bošković Institute, Bijenička c. 54, HR-10000 Zagreb, Croatia*

* *Correspondence: kaizer@almos.uni-pannon.hu; Tel.: +36-88-62 4720.*

Experimental

General remarks

Reactions were carried out in ordinary glassware and chemicals were used as purchased from commercial suppliers without further purification. Synthesis of the ligand was carried out in a microwave reactor (CEM Discover), monitored by TLC on aluminum oxide 60 F_{254} neutral plates and detected with UV lamp (254 nm). Mass spectra were recorded on a HPLC-MS system (Agilent Technologies 1200) coupled with a 6410 Triple-Quadrupole mass spectrometer, operating in a positive ESI mode. NMR spectra were obtained on a Bruker Avance 300 or 600 spectrometer, operating at 300 or 600 MHz for ^{1}H and 75 or 150 MHz for ^{13}C. The spectra are recorded at room temperature. Chemical shifts, δ (ppm), indicate a downfield shift from the residual solvent signal (δ_H: 1.94 ppm, δ_C: 118.26 ppm for CD_3CN and δ_H: 7.26 ppm, δ_C: 77.16 ppm for $CDCl_3$). Coupling constants, *J*, are given in Hz.

Synthesis of ligands 1a-2b, General procedure

The synthesis was performed according to a modified previously reported procedure[1]. The amine (1 eq), K_2CO_3 (12 eq), 2-(chloromethyl)pyridine hydrochloride or 2-(chloromethyl)quinoline hydrochloride (4 eq) and KI (1 eq) were suspended in 50 mL of acetonitrile. The reaction mixture was heated in a microwave reactor (50 W, reflux) for 1 hour. The solvent was evaporated in a vacuum, the residue suspended in ethyl acetate and washed three times with brine and saturated $NaHCO_3$, the organic layer dried over anhydrous sodium sulfate, filtered and evaporated in a vacuum. The crude ligand was purified by automated flash chromatography on a pre-packed neutral alumina column (48 g).

[1] Y. Mikata, Y. Sato, S. Takeuchi, Y. Kuroda, H. Konno and S. Iwatsuki, *Dalton Trans.*, 2013, **42**, 9688–9698.

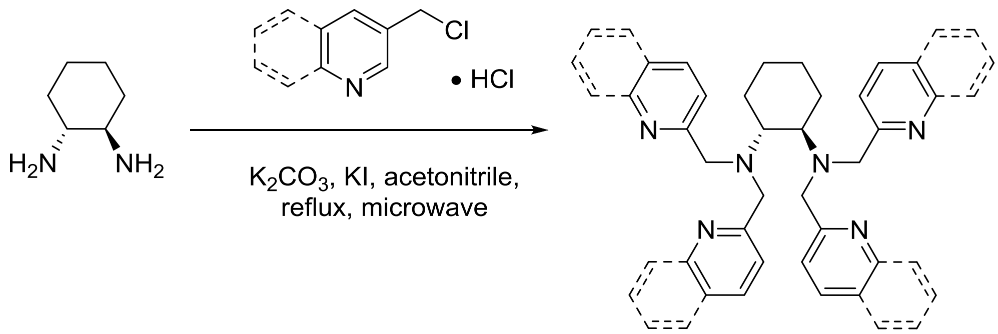

Synthesis of ligands **1a**-**2b (±cda-bpa, and ±cda-bqa)**. Ligands **1a** and **2a** are racemic and ligands **1b** and **2b** are (1R, 2R) isomers.

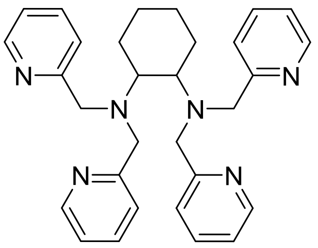

1a, ±cda-bpa

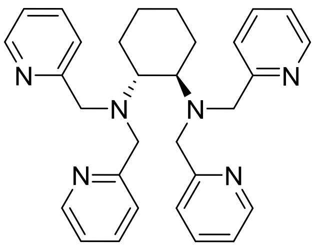

1b, (R, R)-cda-bpa

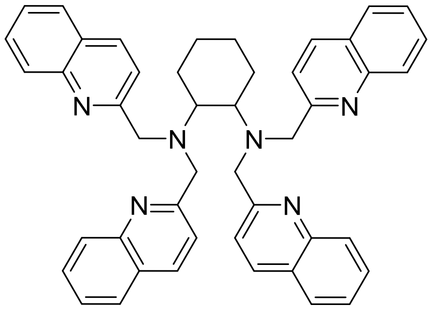

2a, ±cda-bqa

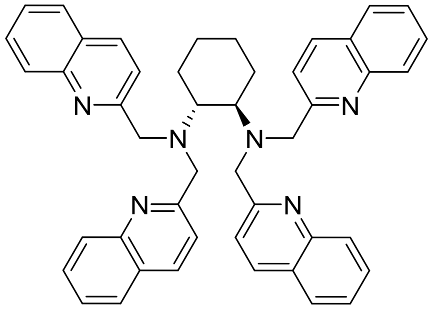

2b, (R, R)-cda-bqa

Figure S1. Ligands 1a (±cda-bpa)-2b (±cda-bqa)

±cda-bpa, 1a

(±)-*trans*-1,2-Diaminocyclohexane (120.0 µL, 1.0 mmol), K_2CO_3 (1658.4 mg, 12.0 mmol), 2-(chloromethyl)pyridine hydrochloride (656.2 mg, 4.0 mmol), KI (166.0 mg, 1.0 mmol). Automated flash chromatography 0 → 5% dichloromethane in methanol (R_f = 0.42, 5 % dichloromethane in methanol). Yield: 303.0 mg (0.6 mmol, 63 %), brown oil.

(R, R)-cda-bpa, 1b

(1*R*,2*R*)-(−)-1,2-Diaminocyclohexane (114.2 mg, 1.0 mmol), K_2CO_3 (1658.4 mg, 12.0 mmol), 2-(chloromethyl)pyridine hydrochloride (656.2 mg, 4.0 mmol), KI (166.0 mg, 1.0 mmol). Automated flash chromatography 0 → 5 % dichloromethane in methanol. Yield: 337.5 mg (0.7 mmol, 71 %), light brown powder.

^{1}H NMR (300 MHz, CD_3CN) δ: 8.39 (d, J = 4.7 Hz, 4H), 7.61 (d, J = 7.8 Hz, 4H), 7.55 – 7.41 (m, 4H), 7.18 – 7.03 (m, 4H), 3.70 (d, J = 14.3 Hz, 4H), 3.58 (d, J = 14.3 Hz, 4H), 2.85 – 2.66 (m, 2H), 1.79 – 1.61 (m, 2H), 1.36 – 1.23 (m, 1H), 1.23 – 1.00 (m, 4H), 0.96 – 0.79 (m, 1H).

^{13}C NMR (75 MHz, CD_3CN) δ: 161.6, 149.5, 136.7, 124.5, 122.6, 60.8, 56.5, 26.8, 25.7.
ESI-MS (*m/z*): 479.2 ($M+H^+$, 100%).

±cda-bqa, 2a

(±)-*trans*-1,2-Diaminocyclohexane (70.5 µL, 0.6 mmol), K_2CO_3 (970.2 mg, 7.0 mmol), 2-(chloromethyl)quinoline hydrochloride (500.0 mg, 2.3 mmol), KI (96.3 mg, 0.6 mmol). No further purification was required, (R_f = 0.28, ethyl acetate:hexane 1:1). Yield: 372.3 mg (0.5 mmol, 94 %), yellow powder.

(R, R)-cda-bqa, 2b

(1*R*,2*R*)-(−)-1,2-Diaminocyclohexane (44.5 mg, 0.4 mmol), K_2CO_3 (651.6 mg, 4.7 mmol), 2-(chloromethyl)quinoline hydrochloride (378.9 mg, 1.6 mmol), KI (64.7 mg, 0.4 mmol). Automated flash chromatography 10 → 40% ethyl acetate in hexane. Yield: 202.3 mg (0.3 mmol, 76 %), light yellow powder.

^{1}H NMR (300 MHz, $CDCl_3$) δ: 7.99 (d, *J* = 8.3 Hz, 4H), 7.91 (d, *J* = 8.5 Hz, 4H), 7.77 (d, *J* = 8.5 Hz, 4H), 7.72 – 7.59 (m, 8H), 7.52 – 7.41 (m, 4H), 4.01 (d, *J* = 14.1 Hz, 4H), 3.85 (d, *J* = 14.1 Hz, 4H), 3.00 – 2.81 (m, 2H), 2.49 – 2.27 (m, 2H), 1.88 – 1.69 (m, 2H), 1.25 – 0.94 (m, 4H).
^{13}C NMR (75 MHz, $CDCl_3$) δ: 161.3, 147.7, 135.8, 129.3, 129.1, 127.6, 127.4, 126.1, 122.2, 60.7, 56.6, 29.8, 26.0, 24.4.
ESI-MS (*m/z*): 679.4 ($M+H^+$, 100%).

ESI-MS of 1

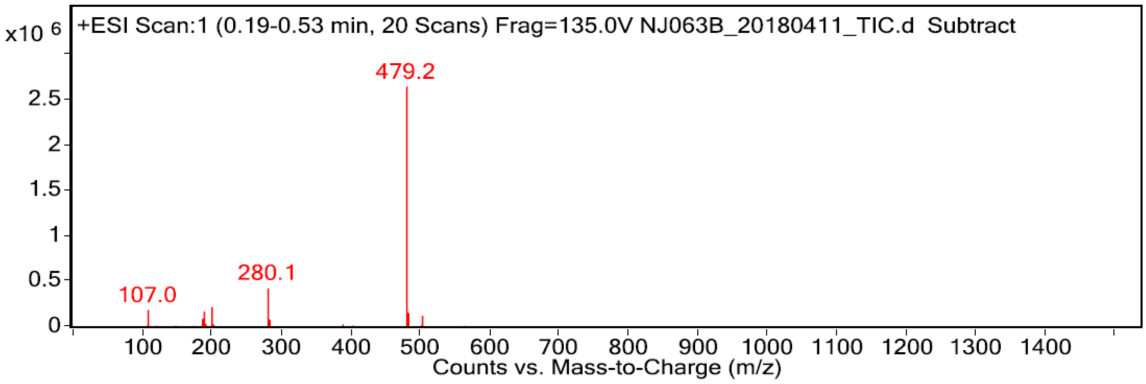

ESI-MS of 2

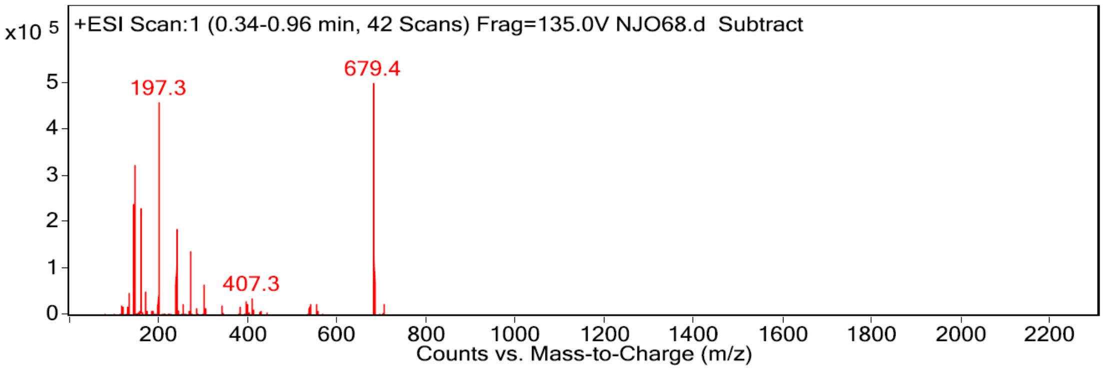

^{1}H NMR (300 MHz, CD_3CN) of **1**

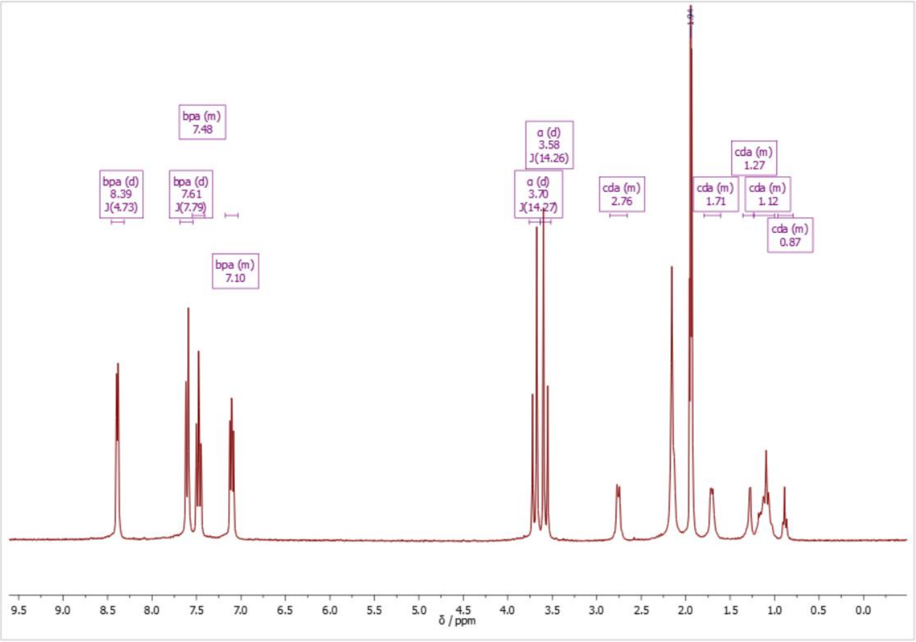

^{13}C NMR (75 MHz, CD_3CN) of **1**

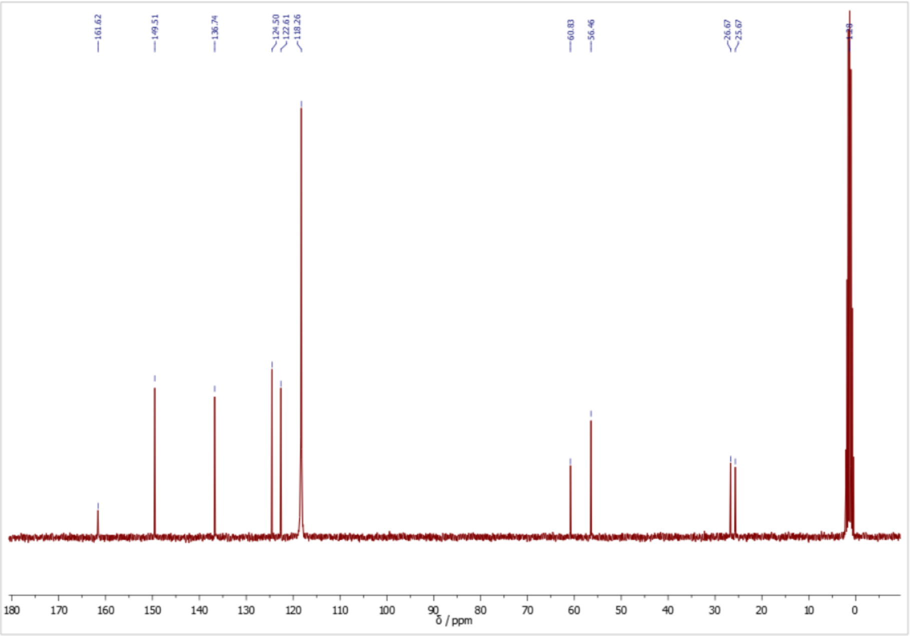

^{1}H NMR (300 MHz, $CDCl_3$) of **2**

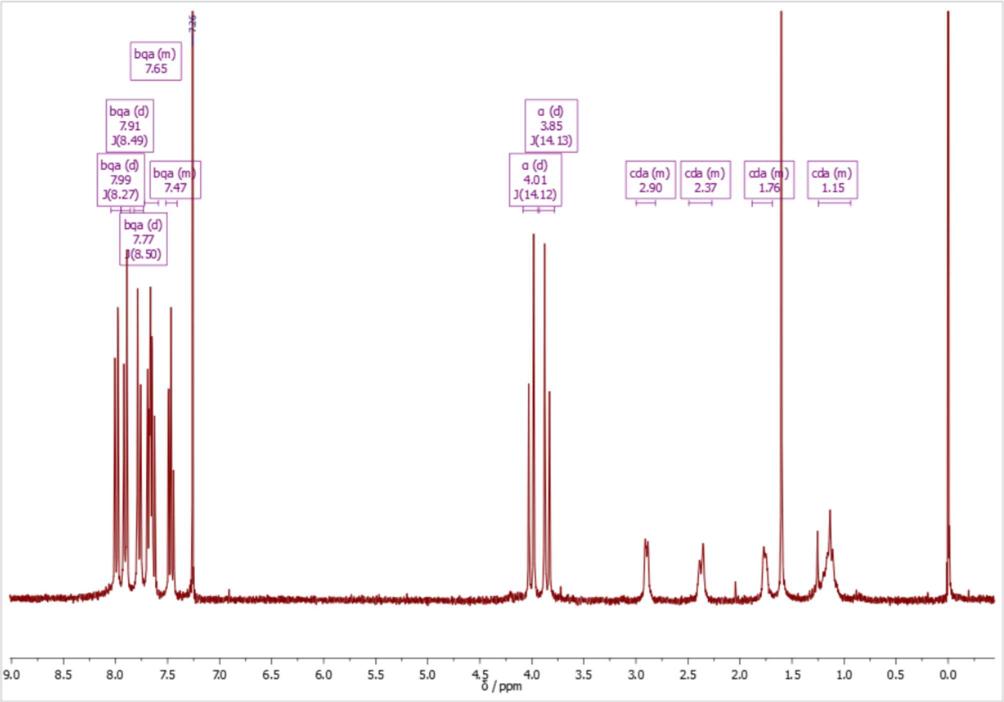

^{13}C NMR (75 MHz, $CDCl_3$) of **2**

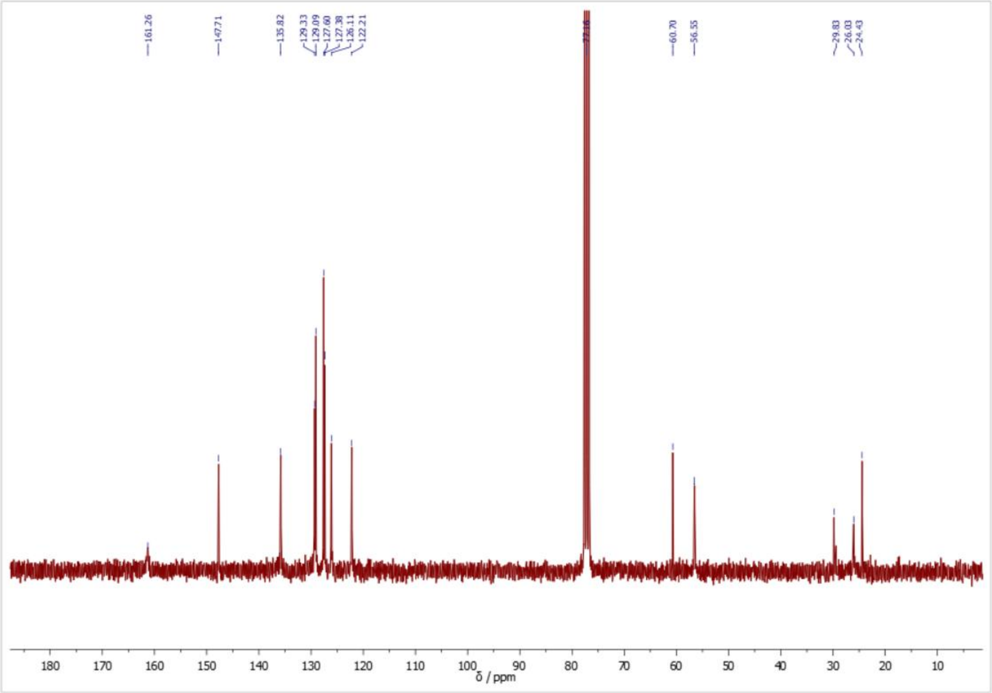

X-ray Crystallography.

Table S1. Experimental data for the X-ray diffraction studies.

**Compound**	***rac*-5**
**Formula**	$C_{50}H_{45}F_6FeN_7O_6S_2$
F_w **(g mol^{-1})**	**1073.90**
**Crystal system**	**Orthorhombic**
**Space group**	***C m c a***
a **(Å)**	**19.7874(9)**
b **(Å)**	**42.5557(15)**
c **(Å)**	**12.4013(4)**
α **(°)**	**90**
**β (°)**	**90**
γ **(°)**	**90**
**V (Å^3)**	**10442.7(7)**
Z	**8**
D_{calc} **(g cm^{-3})**	**1.366**
***F*(000)**	**4432**
**Radiation (Å)**	**1.54184**
**Temperature (*K*)**	**293(2)**
**Reflections collected**	**15441**
**Independent reflections**	**5532**
R_{init}	**0.0314**
**Reflections observed**	**4019**
**Parameters**	**360**
R_1 **[$I > 2\sigma(I)$]** [a]	**0.0761**
wR_2 **(all data)** [b]	**0.2537**
**Goof, *S*** [c]	**1.099**
**Maximum/minimum electron density (e Å^3)**	**0.685/−0.797**

[a] $R_1 = \Sigma||F_o| - |F_c||/\Sigma|F_o|$. [b] $wR_2 = \{\Sigma[w(F_o^2 - F_c^2)^2]/\Sigma[w(F_o^2)^2]\}^{1/2}$. [c] $S = \{\Sigma[w(F_o^2 - F_c^2)^2]/(n - p)\}^{1/2}$ where n is number of reflections and p is the total number of parameters refined.

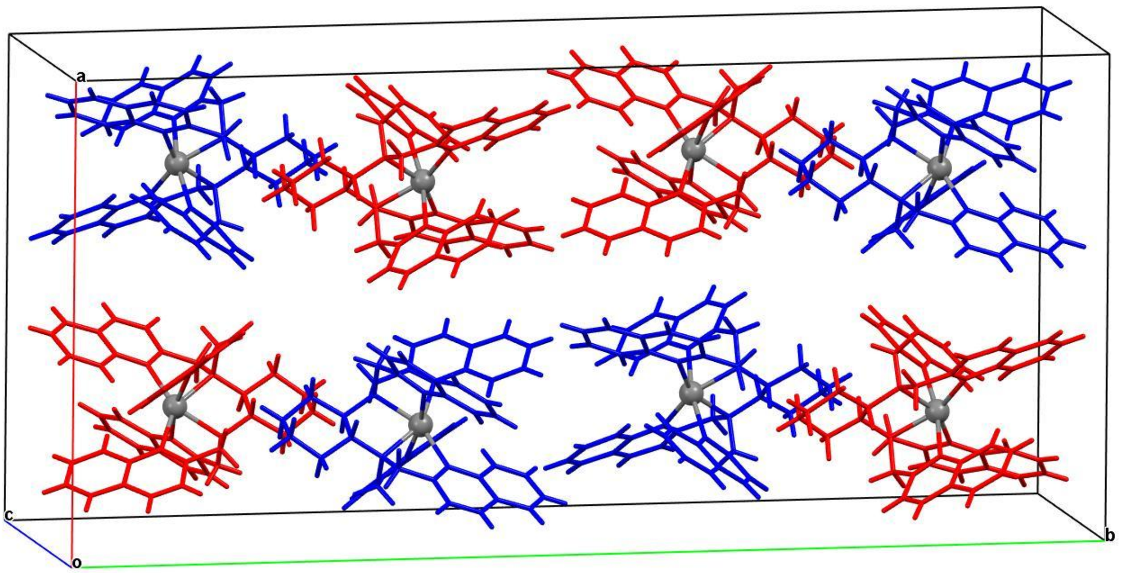

Figure S2. Packing of $[Fe^{II}(CDA\text{-}BQA^*)]^{2+}$ complexes in *rac*-**5**. Individual enetiomers are shown in red and blue colours, respectively. Triflate anions and acetontrile molecules are ommited for clarity.

Table S2. The calculated k_{obs} values in the reaction of **9** and flavanone in MeCN.

N_0	T (K)	$[9]_0$ (10^{-3} M)	Flavanon (M)	k_{obs} ($10^{-2}s^{-1}$)
1	283	2	0.02	1.37±0.05
2	283	2	0.03	1.93±0.09
3	283	2	0.04	2.58±0.13
4	283	2	0.05	3.50±0.14
5	283	2	0.06	4.03±0.16
6	283	2	0.08	5.41±0.19
7	288	2	0.05	4.68±0.16
8	293	2	0.05	5.58±0.20
9	298	2	0.05	6.70±0.22

Table S3. The calculated k_{obs} values in the reaction of **9** and flavanone in MeCN/TFE.

N_0	T (K)	$[9]_0$ (10^{-3} M)	Flavanon (M)	k_{obs} ($10^{-2}s^{-1}$)
1	283	2	0.02	2.64±0.1
2	283	2	0.03	4.28±0.24
3	283	2	0.04	5.99±0.27

Table S4. The calculated k_{obs} values in the reaction of **11** and flavanone in MeCN.

**N_0**	**T (K)**	**$[11]_0$ (10^{-3} M)**	**Flavanon (M)**	**k_{obs} ($10^{-2}s^{-1}$)**
1	283	2	0.03	3.11±0.06
2	283	2	0.04	4.01±0.08
3	283	2	0.05	5.21±0.14
4	283	2	0.10	9.70±0.31

Table S5. The calculated k_{obs} values in the reaction of **10** and flavanone in MeCN/TFE.

**N_0**	**T (K)**	**$[10]_0$ (10^{-3} M)**	**Flavanon (M)**	**k_{obs} ($10^{-2}s^{-1}$)**
1	283	2	0.02	0.848±0.02
2	283	2	0.03	1.27±0.04
3	283	2	0.04	1.59±0.05
4	283	2	0.05	2.07±0.06
5	288	2	0.05	1.30±0.03
6	293	2	0.05	1.67±0.04
7	298	2	0.05	2.28±0.08
